# Molecular Determinants and Pharmacological Analysis for a Class of Competitive Non-transported Bicyclic Inhibitors of the Betaine/GABA Transporter BGT1

**DOI:** 10.3389/fchem.2021.736457

**Published:** 2021-09-14

**Authors:** Stefanie Kickinger, Maria E. K. Lie, Akihiro Suemasa, Anas Al-Khawaja, Koichi Fujiwara, Mizuki Watanabe, Kristine S. Wilhelmsen, Christina B. Falk-Petersen, Bente Frølund, Satoshi Shuto, Gerhard F. Ecker, Petrine Wellendorph

**Affiliations:** ^1^ Department of Drug Design and Pharmacology, Faculty of Health and Medical Sciences, University of Copenhagen, Copenhagen, Denmark; ^2^ Department of Pharmaceutical Science, University of Vienna, Vienna, Austria; ^3^ Faculty of Pharmaceutical Sciences, Hokkaido University, Sapporo, Japan

**Keywords:** SLC6 neurotransmitter transporters, competitive inhibition, binding mode analysis, computational chemistry, bicyclo-GABA, BGT1, GABA transporter, molecular docking, molecular dynamics

## Abstract

The betaine/GABA transporter 1 (BGT1) is a member of the GABA transporter (GAT) family with still elusive function, largely due to a lack of potent and selective tool compounds. Based on modeling, we here present the design, synthesis and pharmacological evaluation of five novel conformationally restricted cyclic GABA analogs related to the previously reported highly potent and selective BGT1 inhibitor (1*S*,2*S*,5*R*)-5-aminobicyclo[3.1.0]hexane-2-carboxylic acid (bicyclo-GABA). Using [^3^H]GABA radioligand uptake assays at the four human GATs recombinantly expressed in mammalian cell lines, we identified bicyclo-GABA and its *N*-methylated analog (**2**) as the most potent and selective BGT1 inhibitors. Additional pharmacological characterization in a fluorescence-based membrane potential assay showed that bicyclo-GABA and **2** are competitive inhibitors, not substrates, at BGT1, which was validated by a Schild analysis for bicyclo-GABA (p*K*_*B*_ value of 6.4). To further elaborate on the selectivity profile both compounds were tested at recombinant α_1_β_2_γ_2_ GABA_A_ receptors. Whereas bicyclo-GABA showed low micromolar agonistic activity, the *N*-methylated **2** was completely devoid of activity at GABA_A_ receptors. To further reveal the binding mode of bicyclo-GABA and **2** binding hypotheses of the compounds were obtained from *in silico*-guided mutagenesis studies followed by pharmacological evaluation at selected BGT1 mutants. This identified the non-conserved BGT1 residues Q299 and E52 as the molecular determinants driving BGT1 activity and selectivity. The binding mode of bicyclo-GABA was further validated by the introduction of activity into the corresponding GAT3 mutant L314Q (38 times potency increase cf. wildtype). Altogether, our data reveal the molecular determinants for the activity of bicyclic GABA analogs, that despite their small size act as competitive inhibitors of BGT1. These compounds may serve as valuable tools to selectively and potently target BGT1 in order to decipher its elusive pharmacological role in the brain and periphery such as the liver and kidneys.

## Introduction

In the mammalian brain, γ-aminobutyric acid (GABA) functions as the principal inhibitory neurotransmitter. GABA’s inhibitory effect is mediated through ionotropic or metabotropic GABA receptors. The ionotropic GABA type A (GABA_A_) receptors are located either in the synapse where they mediate phasic inhibition, or extrasynaptically, where they mediate tonic inhibition (Olsen and Sieghart, 2009). As members of the GABAergic system, the GABA transporters (GAT) are key for regulating both phasic and tonic inhibition by facilitating the uptake of GABA together with sodium and chloride ions into the presynaptic neurons and surrounding glial cells (Farrant and Nusser, 2005). Four different GAT subtypes have been identified: GAT1, GAT2, GAT3 and betaine/GABA transporter 1 (BGT1) corresponding to the human genome nomenclature SLC6A1, SLC6A13, SLC6A11 and SLC6A12, respectively (Hediger et al., 2004). Imbalances in GABAergic signaling as a result of altered GABA uptake have been linked to several neurological disorders e.g. epilepsy, Alzheimer’s disease and ischemia (Clarkson et al., 2010; Wu et al., 2014; Lie et al., 2017). In the last decades, a plethora of efforts was spent to selectively inhibit GATs pharmacologically, especially GAT1 due to its high expression level in the brain, resulting in the antiepileptic drug tiagabine (Gabitril®) (Nielsen et al., 1991). Substantial side effects related to tiagabine treatment (Kälviäinen, 2001, 2002; Schousboe et al., 2011) have shifted the scientific focus towards non-GAT1 subtypes including BGT1 (Damgaard et al., 2017). Although BGT1 is expressed in scarce amounts in the brain, BGT1 inhibition has been proposed as a novel therapeutic strategy to treat epileptic seizures due to its extrasynaptic localization and reported anti-seizure actions (White et al., 2005; Vogensen et al., 2013; Schousboe et al., 2017). Noteworthily, high levels of BGT1 are expressed in the periphery in the liver and the kidneys (Zhou et al., 2012) where it is believed to constitute a link between osmotic stress and chronic inflammatory diseases (Kempson et al., 2014). In the kidneys, BGT1 accumulates betaine in medullary cells and therefore facilitates osmoprotection, while in the liver it is proposed to play a role for liver metabolism by providing betaine as a methyl donor for the betaine-homocysteine S-methyltransferase (Zhou et al., 2012; Kempson et al., 2014). Interestingly, studies in BGT1 knock-out mice showed no altered seizure susceptibility nor intolerance to salt treatment (Lehre et al., 2011; Zhou et al., 2012). Thus, to further decipher the physiological and pharmacological relevance of BGT1, selective and potent BGT1 inhibitors with fully characterized inhibition profiles are imperative.

**FIGURE 1:**
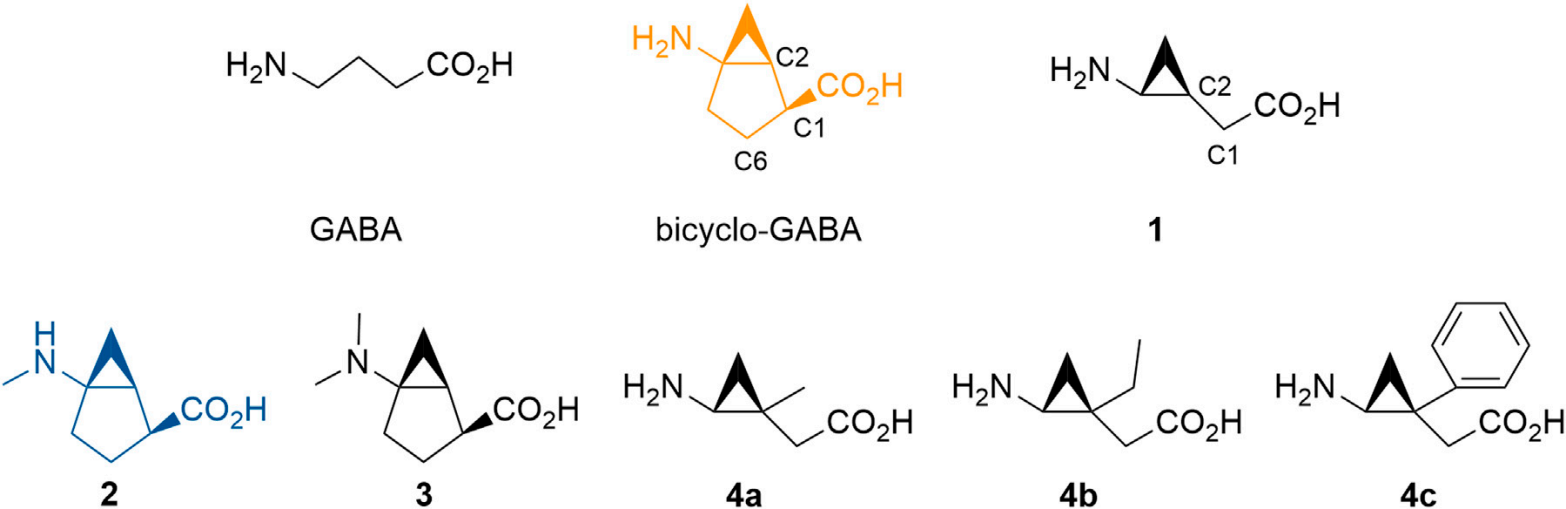
GABA and novel analogs of bicyclo-GABA and **1** used in this study. The color code for bicyclo-GABA (orange) and **2** (blue) is applied throughout the manuscript.

Although achieving subtype-selective inhibition of BGT1 is challenged by the high sequence identity with GAT2/3 within the orthosteric binding pocket (Kickinger et al., 2019), a strategy based on conformational restriction using cyclopropane as a key structure was devised (Mizuno et al., 2017). Hence, the cyclic GABA analog (1*S*,2*S*,5*R*)-5-aminobicyclo[3.1.0]hexane-2-carboxylic acid (bicyclo-GABA, Figure 1) was designed and synthesized as the first selective BGT1 inhibitor with high nanomolar activity (IC_50_ 590 nM) (Kobayashi et al., 2014). This compound was the result of conformationally restricting (2*S*,3*R*)-4-amino-3,4-methanobutyric acid (**1**) (Figure 1) with a rigid bicyclo[3.1.0]hexane backbone. The *syn* conformation of **1** and bicyclo-GABA was identified as the bioactive conformation, as bicyclo-GABA showed a 9 times higher activity for BGT1 compared to **1**. Additionally, the conformational restriction of **1** into bicyclo-GABA dramatically increased subtype-selectivity, as bicyclo-GABA displayed 129 times higher activity for BGT1 than GAT3 compared to a modest three times higher activity for **1**. Still, the molecular determinants and exact pharmacological profile for the compound class represented by bicyclo-GABA remain elusive. In this study, guided by molecular docking and molecular dynamics (MD) studies, we designed, synthesized and pharmacologically characterized five novel cyclic GABA derivatives (Figure 1). Furthermore, through chemical design, it was attempted to reduce off-target effects of the compound series at GABA_A_ receptors, another important brain-relevant GABA target. From the computational modeling, potential residues involved in the BGT1 interaction were identified and verified by site-directed mutagenesis. Additionally, to investigate the precise mode of inhibition, a fluorometric imaging plate reader (FLIPR) membrane potential (FMP) functional cell-based assay for BGT1 was applied. Overall, the study presents the experimentally validated binding modes of synthetically optimized bicyclic GABA analogs as BGT1-selective non-transported competitive inhibitors as important new tool compounds to improve our understanding of the biological relevance of BGT1.

## Results

### Identification of the Binding Mode of Bicyclo-GABA by Computational Modeling

The finding that cyclic GABA analogs typified by bicyclo-GABA inhibit BGT1 in a stereospecific and relatively potent manner (Kobayashi et al., 2014) prompted us to perform molecular docking and MD simulations to guide medicinal chemistry.

**FIGURE 2:**
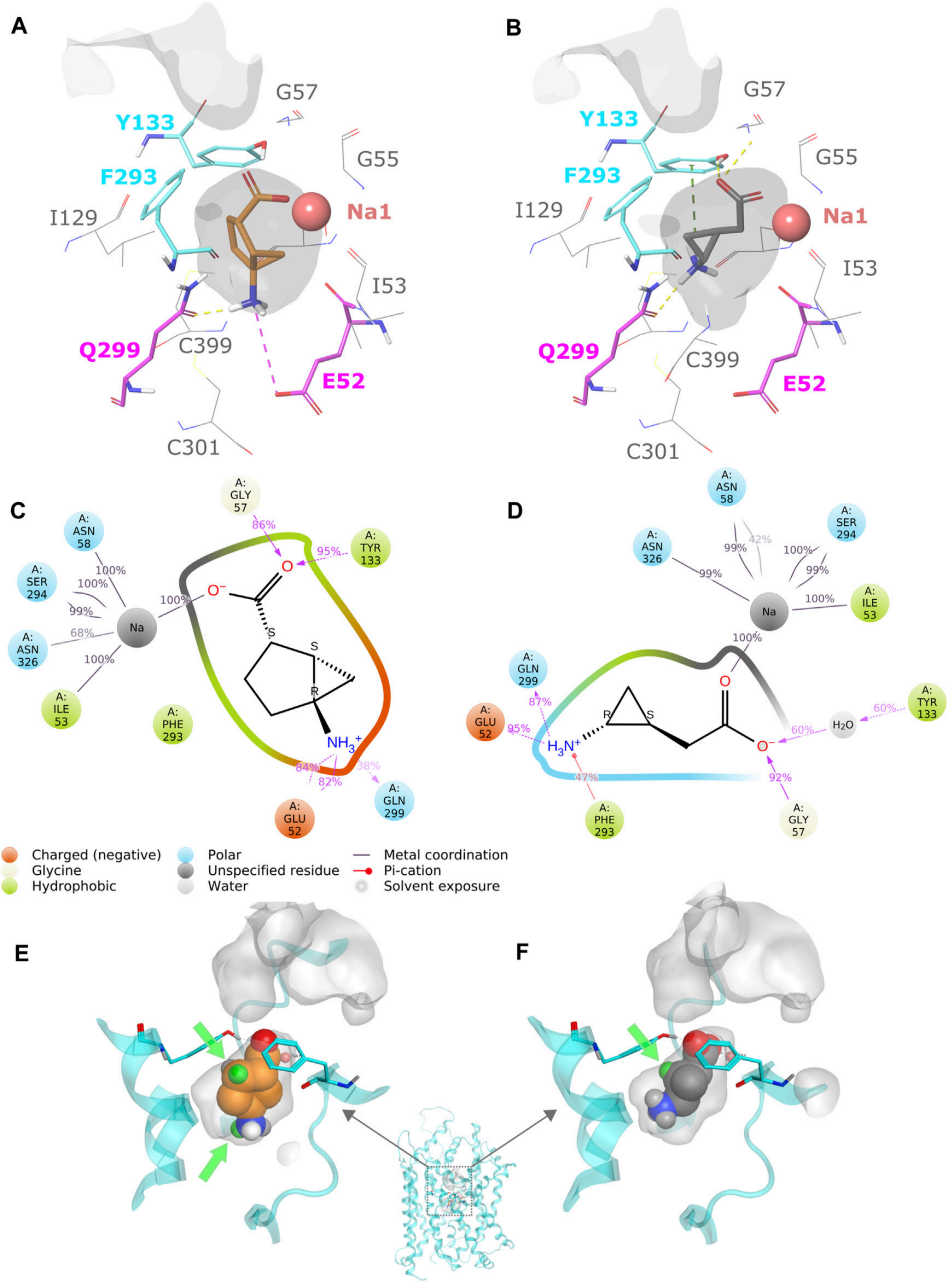
**(A)** Protein–ligand interactions of the most promising docking pose of bicyclo-GABA. The residues E52 and Q299 are highlighted in magenta and the extracellular lid residues F293 and Y133 are highlighted in cyan. The pocket surface is depicted in grey. **(B)** Protein–ligand interactions of the most promising docking pose of **1**. **(C)** Protein–ligand interaction schematic overview of bicyclo-GABA after 100 ns of MD simulations (see also Supplementary Figure S3). **(D)** Protein–ligand interaction schematic overview of **1** after 100 ns of MD simulations (see also Supplementary Figure S4). **(E)** Available pocket space for the docking pose of bicyclo-GABA. The pocket surface is depicted in grey. Green spheres represent the selected derivatization positions. The extracellular lid residues are depicted in cyan. **(F)** Available pocket space of the docking pose of **1**.

As bicyclo-GABA is a conformationally restricted small analog of GABA, we inferred that it would function as a competitive substrate inhibitor interacting with the orthosteric site of the transporter. In order to identify the molecular determinants governing the inhibitory activity of bicyclo-GABA, we first performed induced-fit docking (Sherman et al., 2006) of bicyclo-GABA as well as the cyclopropane-based analog **1** into the orthosteric binding site of a human BGT1 homology model (Damgaard et al., 2015) (for pocket definition see methods section and Supplementary Table S1). We chose to dock the compounds into an outward-occluded conformation due to the small size of the compounds and their anticipated competitive inhibition profile (Kickinger et al., 2019). All poses were analyzed according to their docking scores (Glide gscores and emodel scores) (Friesner et al., 2004), and additionally clustered according to their common scaffold to get an overview of the possible orientations of bicyclo-GABA and **1** in the pocket (Supplementary Figures S1, S2). Among the top-scored poses, both compounds showed a similar binding mode. The carboxyl group of bicyclo-GABA and **1** coordinates the sodium (Na1) in the pocket, which is a key feature of GABA transport inhibition (Figures 2A,B) (Skovstrup et al., 2010, 2012; Kristensen et al., 2011; Jurik et al., 2015; Vogensen et al., 2015; Kickinger et al., 2019). The protonated nitrogen of bicyclo-GABA and **1** undergoes hydrogen bonding with the sidechain of Q299 and either forms a salt bridge with E52 (with bicyclo-GABA) or a π-cation interaction with Y133 (with **1**). In order to check the stability of the most promising binding poses of bicyclo-GABA and **1**, MD simulations of 100 ns were performed. The simulations confirmed that the carboxyl group of both compounds coordinates Na1 and also involves hydrogen bonding with G52 and the sidechain of Y133. Moreover, the protonated nitrogen of both compounds undergoes hydrogen bonding with the sidechain of Q299 as well as hydrogen bonding with the sidechain of E52 (Figures 2C,D as well as Supplementary Figures S3, S4). The π-cation interaction between the protonated nitrogen of **1** and Y133 observed in the docking study was not present in the simulations.

### Design of Novel Cyclic GABA Analogs

Based on the binding hypothesis of bicyclo-GABA and **1**, we visually analyzed the available space in the binding pocket to rationalize possible sites for chemical derivatization. The aim of the derivatization was twofold: 1) to facilitate blood-brain-barrier permeability by introducing lipophilic substituents; a strategy successfully applied to several known GAT inhibitors such as tiagabine, EF1502 and RPC-425 (Skovstrup et al., 2010; Vogensen et al., 2013; Lie et al., 2018, 2020; Kickinger et al., 2019), and 2) to identify positions for derivatization that would reduce off-target effects at GABA_A_ receptors, most particularly α_1_β_2_γ_2_ receptors, the major GABA_A_ receptor subtype in the brain (Olsen and Sieghart, 2009).

According to our analysis based on the docking results, the carbon 6 (C6) position of bicyclo-GABA (Figure 1) was identified as the most promising position for derivatization since it is located next to the extracellular lid (Y133, F293) (Figure 2E), which would allow bulky substituents to expand into the extracellular vestibule. Introduction of substituents at this position was initially attempted using Pd(II)-catalyzed C(sp^3^)–H alkylation using a substrate, which had a directing group at the carboxy group of a derivative of bicyclo-GABA. However, we soon realized that introduction of substituents at this unfunctionalized sp^3^-carbon in the bicyclic ring system was synthetically unfeasible. Since previous reports suggest that ligands for the orthosteric binding site at GABA_A_ receptors are sensitive to steric hindrance in proximity of the cationic moiety of the molecules, we instead focused on improving selectivity *via N*-alkylation of bicyclo-GABA (Krogsgaard-Larsen and Johnston, 1978; Haefliger et al., 1984; Falch et al., 1986; Petersen et al., 2014). Given the limited space predicted by the homology model (Figure 2E), only small substituents such as methyl were included. Hence, the derivatives **2** (methyl) and **3** (dimethyl) of bicyclo-GABA were designed (Figure 1).

To further probe the space available for bulky substituents in the BGT1 binding pocket, we turned to **1** which, albeit being 9 times less potent than bicyclo-GABA (Kobayashi et al., 2014), is synthetically better apt for chemical derivatization. In a previous study, exploring substitution at carbon 1 (C1) of **1** (Figure 1), we showed that only small substituents such as methyl groups are tolerated (Suemasa et al., 2018). In line with docking studies, we identified carbon 2 (C2) (Figure 1) as a potential additional site for derivatization because it is predicted to be located close to the extracellular lid in our docking poses, thus allowing room for bulky substituents in the extracellular vestibule (Figure 2F). Consequently, derivatives substituted at C2 with methyl (**4a)**, ethyl (**4b)** and phenyl (**4c**) groups were designed (Figure 1).

### Chemical Synthesis of Novel Cyclic GABA Analogs

**FIGURE 3:**
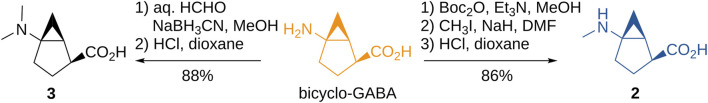
Synthesis of *N*-methylated bicyclic GABA analogs.

**FIGURE 4:**
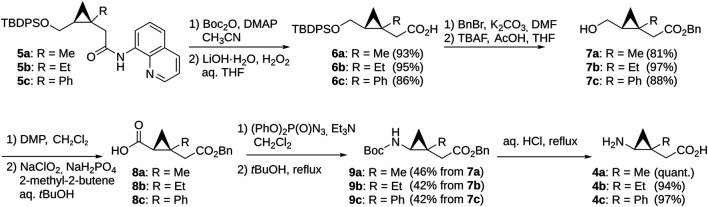
Synthesis of GABA analogs containing the trisubstituted cyclopropane backbone.

The *N*-substituted derivatives **2** and **3** were prepared *via* standard alkylation or reductive amination from bicyclo-GABA (Figure 3) whereas the synthesis of **4a–c,** containing the chiral trisubstituted cyclopropane structure including a quaternary carbon stereocenter, was troublesome. However, we recently developed new methods for constructing the chiral cyclopropane structures by Pd(II)-catalyzed tertiary C(sp^3^)–H arylation *via* directing group-mediated C–H activation (Hoshiya et al., 2013, 2016, 2017; Minami et al., 2019). Using this method, we constructed **5a–c** (Supplementary Figure S8). This prompted the synthesis of the desired **4a–c** because **5a–c** were effective precursors for synthesizing the desired compounds **4a–c**. Synthesis of the target compounds **4a–c** is shown in Figure 4. The aminoquinoline group of chiral trisubstituted cyclopropanes **5a–c** were removed by a two-step method to give **6a–c**. After protecting group manipulation of **6a–c** giving **7a–c**, successive oxidation of the hydroxymethyl moiety provided the carboxylic acids **8a–c**. Azidation of **8a–c** and subsequent treatment under refluxing conditions in *t*-BuOH afforded the Curtius rearrangement products **9a–c**. Acidic treatment of **9a–c** for simultaneously removing the benzyl and Boc protecting groups finally provided the target compounds **4a–c**.

### Initial Pharmacological Characterization and Docking of Novel Derivatives

**FIGURE 5:**
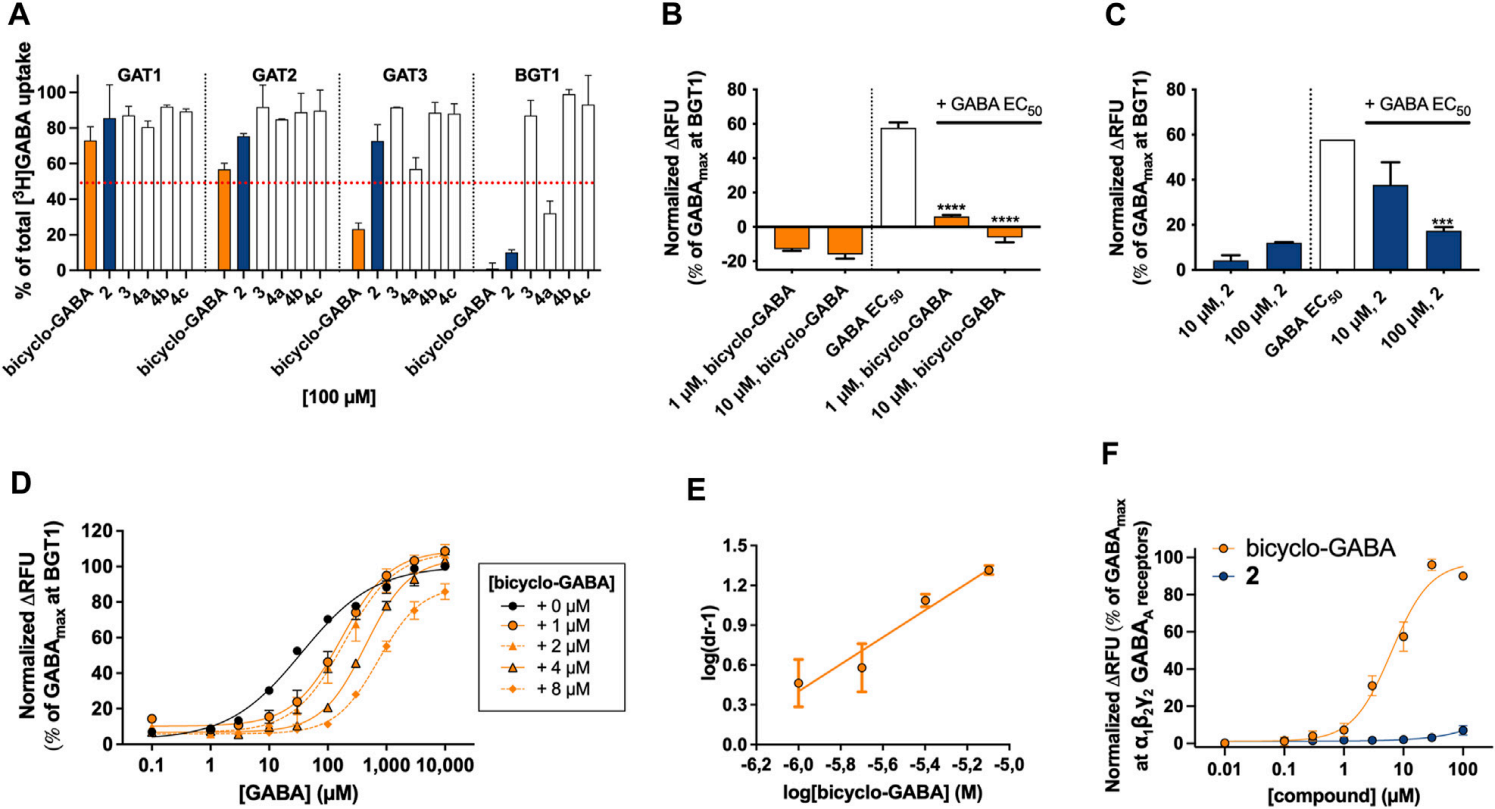
**(A)** Pharmacological analysis of cyclic GABA analogs in the [^3^H]GABA uptake assay at the four human GATs. Percentage inhibition of [^3^H]GABA uptake by analogs at 100 µM concentrations. Data shown are pooled and normalized to 100% [^3^H]GABA uptake with the dashed red line indicating 50% inhibition (*n* = 2). **(B**–**C)** Inhibitory, but not substrate, activity of the two most potent bicyclo-GABA analogs at BGT1 in the FMP assay. Data shown are pooled and normalized responses (% of GABA_max_ at BGT1) of **(B)** bicyclo-GABA (n = 4) and **(C)** **2** (*n* = 2) without and with GABA EC_50_ (25 µM GABA) present. **(D**–**E)** Gaddum-Schild analysis of bicyclo-GABA, yielding a Schild slope of 1.02 indicative of competitive inhibition with a calculated *K*_*B*_ of 0.47 µM (*n* = 4). **(F)** Characterization of bicyclo-GABA and **2** at α_1_β_2_γ_2_ GABA_A_ receptors by use of the FMP assay. Data in **B(**–**C)** were compared to GABA EC_50_ by a One-way ANOVA followed by Dunnett’s multiple comparison test, significance levels ***p < 0.001, ****p < 0.0001. Data are presented as means ± S.E.M.

To initially evaluate the activities of the new cyclic/bicyclic GABA analogs, the compounds were tested at a single concentration of 100 µM in a previously described cell-based [^3^H]GABA uptake assay at each of the four human GABA transporters (Al-Khawaja et al., 2014). Here, only **2 (**90% inhibition**)** and **4a (**68% inhibition**)** showed noticeable activity at BGT1, whereas **3**, **4b,** and **4c** showed no or negligible activity (<15% inhibition) at any of the GATs (Figure 5A). Based on this finding, only **2** and bicyclo-GABA were further investigated pharmacologically.

Docking of all suggested derivatives was performed and used to rationalize the observed activities in the initial pharmacological screening. The docking poses of all compounds were again clustered according to the common scaffold including the poses of bicyclo-GABA and **1** (Supplementary Figure S5). We observed that **2**, **3**, **4a** and **4b** were able to adopt a similar binding orientation as bicyclo-GABA within the most populated cluster and should therefore be able to also undergo interactions with Q299 and E52 (see interaction fingerprint analysis in Supplementary Figure S6). No poses of **4c** were found in the most prominent docking pose cluster suggesting that **4c** cannot adopt the same binding orientation due to the bulky substituent (Supplementary Figure S5). Thus, we predicted that derivatives of **1 (4a–c)** would show decreased activity with increasing substituent size.

### Pharmacological Characterization of the Inhibition Profile of Bicyclo-GABA and 2

In order to scrutinize the mode of inhibition of bicyclo-GABA and the *N*-methylated derivative **2**, the ability to be translocated by BGT1 was investigated in the functional FMP assay able to discriminate between substrates and inhibitors, as previously described (Al-Khawaja et al., 2014). Interestingly, when tested as substrates, no increase in fluorescence signals in response to either bicyclo-GABA or **2** was observed, demonstrating that neither of them are transported by BGT1 (Figures 5B,C). A similar lack of activity was observed at GAT1 (Supplementary Figure S7). Accordingly, when testing both compounds as inhibitors, in the presence of a GABA EC_50_ concentration (25 µM) at BGT1, both compounds behaved as inhibitors (Figures 5B,C). This experiment further highlights a relatively higher potency of bicyclo-GABA compared to the *N*-methyl analog **2**, with the latter having an approximate IC_50_ value of 10 µM. Further, to rule out any potential quenching of the fluorescence for this class of compounds, we tested bicyclo-GABA at GAT1 in parallel in the presence of a GABA EC_50_ concentration (10 µM) (Supplementary Figure S5). From this it is clear that the inhibitory effect of bicyclo-GABA is specific to BGT1. To further probe the mode of inhibition of bicyclo-GABA at BGT1, we performed a Gaddum-Schild analysis by co-applying bicyclo-GABA at different fixed concentrations with increasing concentrations of GABA (Figure 5D). Consistent with a simple competitive profile of inhibition, we observed significant rightward shifts of the GABA curves in the presence of increasing concentrations of bicyclo-GABA, while R_max_ was overall unchanged. Only at the highest concentration of bicyclo-GABA did we observe a significantly decreased maximum response (Figure 5D; Table 1). The Gaddum-Schild plot further revealed a linear regression with a slope of 1.02 (Figure 5E), which confirms the competitive profile (*K*_*B*_ value of 0.47 µM; p*K*_*B*_ ± S.E.M: 6.4 ± 0.09).

**TABLE 1:** The effect of increasing concentrations of the inhibitor bicyclo-GABA on the GABA EC_50_ and R_max_ at BGT1 measured in the FMP assay. The GABA EC_50_ and R_max_ values are compared to the corresponding values in the absence of bicyclo-GABA by a One-way ANOVA followed by Dunnett’s multiple comparison test, significance levels *p < 0.05, ***p < 0.001, ****p < 0.0001.

[Bicyclo-GABA] (µM)	EC_50_ (µM) (pEC_50_ ± S.E.M.)	R_max_ ± S.E.M. (%)
0	36 (4.4 ± 0.03)	100
1	172 (3.8 ± 0.14)***	109 ± 0.6
2	217 (3.7 ± 0.15)***	106 ± 5.5
4	450 (3.3 ± 0.02)****	103 ± 3.4
8	738 (3.1 ± 0.02)****	86 ± 4.4*

### Evaluation of Activity at α_1_β_2_γ_2_ GABA_Α_ Receptors

Since bicyclo-GABA and **2** are GABA analogs, we wanted to further investigate whether these compounds show activity at a GABA target unrelated to transporters e.g. GABA_A_ receptors, and whether substitution of the *N*-methyl group affects this. For this purpose we chose the major GABA_A_ receptor subtype in the brain, α_1_β_2_γ_2_, and used the FMP assay for functional assessment as previously reported (Falk-Petersen et al., 2020). This assay detects changes in membrane potential for an average cell population through changes in fluorescence of a membrane pre-equilibrated fluorescent blue dye. It therefore provides an indirect measure of activity with the advantage of being able to discern between GAT substrates and inhibitors (Kragholm et al., 2013; Al-Khawaja et al., 2014; Kickinger et al., 2020).

While **2** was indeed inactive at 100 μM, bicyclo-GABA displayed low micromolar activity at α_1_β_2_γ_2_ GABA_A_ receptors (EC_50_ = 5.1 µM; pEC_50_ ± S.E.M: 5.3 ± 0.10) (Figure 5F). These results demonstrate the successful identification of **2** as a BGT1 inhibitor devoid of activity at a prototypical GABA_A_ receptor albeit with some loss of potency and selectivity towards BGT1.

### Validation of the Binding Mode by Site-Directed Mutagenesis

**FIGURE 6:**
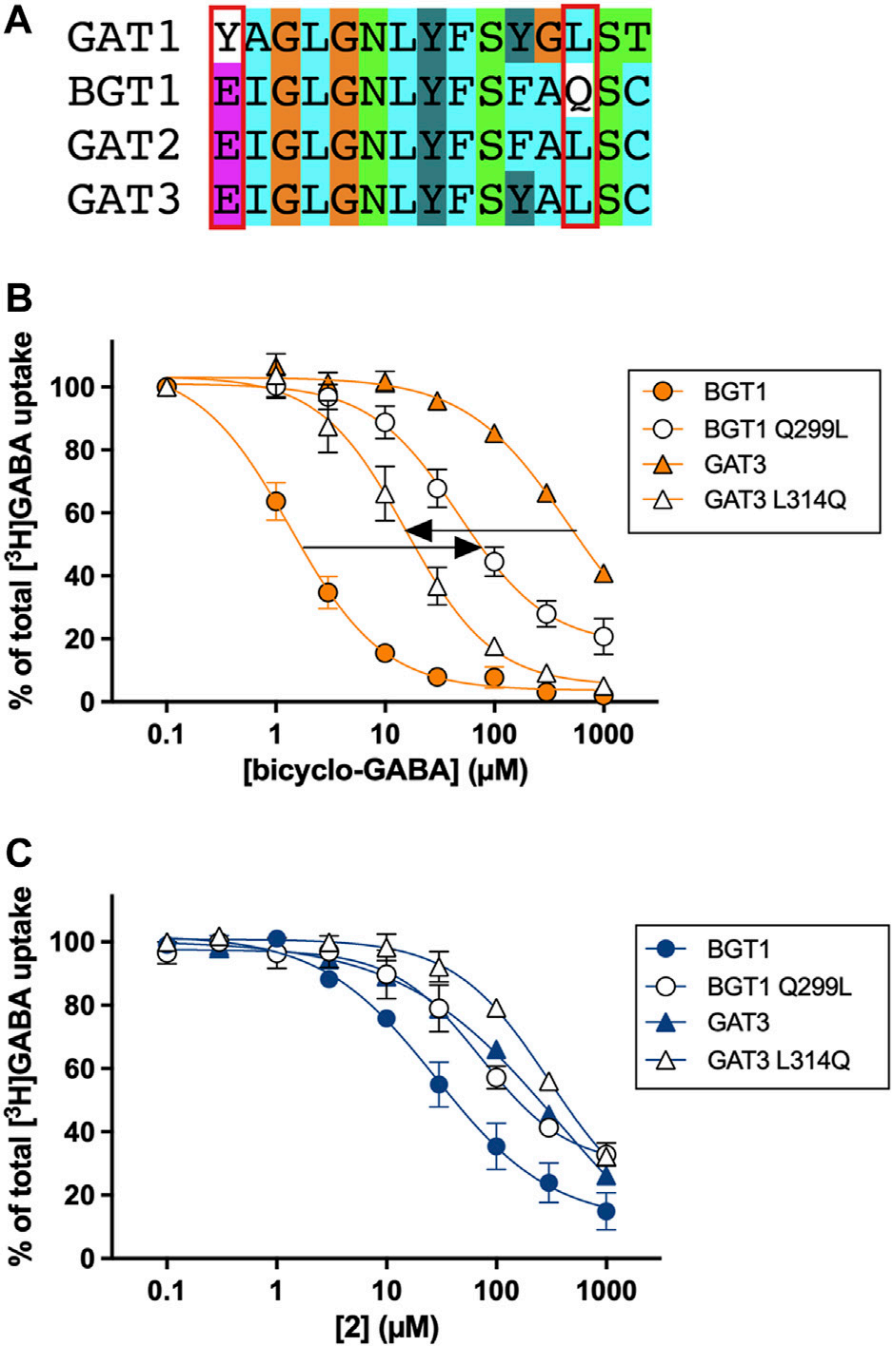
**(A)** Pocket alignment of the orthosteric site of the four GAT subtypes colored according to the default ClustalX color scheme (Larkin et al., 2007). The pocket was defined as all corresponding GAT residues (15 in total) that are within a distance of 4.5 Å of the co-crystallized ligand leucine in the homologous LeuT crystal structure (PDB ID 2A65) (Yamashita et al., 2005) according to the alignment by Kickinger *et al.* (Kickinger et al., 2019). The mutated residues in BGT1 and the corresponding residues in GAT1-3 are indicated by a red box. The numbering of all residues within the orthosteric pocket can be found in the Supplementary Table S1. [^3^H]GABA uptake concentration-response curves of **(B)** bicyclo-GABA and **(C)** **2** at BGT1 (wt), BGT1 Q299L, GAT3 (wt) and GAT3 L314Q transiently expressed in tsA201 cells (human subtypes). Pooled data (n = 3–4) based on technical triplicates, normalized to 100% [^3^H]GABA uptake (presented as means ± S.E.M). The IC_50_ and pIC_50_ values are presented in Table 2.

To experimentally validate the proposed binding mode of bicyclo-GABA and its most active derivative **2**, we performed site-directed mutagenesis studies. According to the docking and MD results, the sidechains of Q299 and E52 were identified to form crucial interactions with the protonated nitrogen of both compounds. The residue Q299 in human BGT1 corresponds to leucine in all other GATs (human, rat, mouse), while E52 corresponds to tyrosine in GAT1 (human, rat, mouse) but is conserved among GAT2, GAT3 and BGT1 (human, rat, mouse) (Figure 6A) (Kickinger et al., 2019). Thus, we engineered the corresponding mutants Q299L and E52Y in human BGT1 and predicted that the introduction of these mutations would lead to decreased activity of both bicyclo-GABA and **2** due to the loss of essential hydrogen bond interactions. Furthermore, we wanted to test the double mutant E52Y + Q299L. However, in a previous study this mutant was proven non-functional (Kickinger et al., 2020). Instead, we generated the double mutant E52A + Q299L as well as the single mutant E52A. For these mutants we predicted decreased activity. Lastly, the reciprocal mutation to BGT1 Q299L was created in GAT3 (L314Q), ideally expecting a gain-of-function.

The experimental results of this systematic testing obtained using the cell-based [^3^H]GABA uptake assay are illustrated in Figures 6B,C, and average data are collected in Table 2. As inferred from the initial testing (Figure 5), **2** was found to be a good inhibitor of BGT1 albeit with a lower IC_50_ (9.8 µM) than bicyclo-GABA (1.5 µM). Further, **2** displayed decreased selectivity for BGT1 over GAT3 (selectivity ratio of 22) compared to bicyclo-GABA (selectivity ratio of 407) (Figure 6B; Table 2). When testing the mutants postulated to be important for binding, we observed that the inhibitory activity of bicyclo-GABA was significantly decreased at the BGT1 mutants containing either the single mutants Q299L (32 times) and E52Y (7 times) or the double mutant Q299L + E52A (27 times) compared to wildtype (wt) (Figure 6B; Table 2). The same pattern was observed for **2** although less pronounced. By contrast, GABA showed increased activity across the different mutants. This is in line with the general observation that GABA shows higher activity at GAT1-3 (Kickinger et al., 2019). Interestingly, for the E52A mutant, both bicyclo-GABA and **2** presented a remarkable and consistent significant increase in activity (38 and 82 times respectively; Table 2). For bicyclo-GABA, a quite dramatic increase in inhibitory activity was obtained (IC_50_ value of 40 nM). By contrast, GABA activity was not significantly altered in this mutant. Finally, most remarkably, the GAT3 mutant L314Q resulted in a significantly increased inhibitory activity for bicyclo-GABA (38 times) and GABA (1.6 times) relative to wt, however the activity was not increased for **2** (Figures 6B,C; Table 2). Altogether, these data support the central involvement of Q299 and E52 for the activity and selectivity of bicyclic GABA analogs at BGT1.

**TABLE 2:** Inhibitory activities of GABA, bicyclo-GABA and **2** tested in the [^3^H]GABA uptake assay at BGT1, GAT3 and selected mutants transiently expressed in tsA201 cells. Data represent at least three independent experiments in triplicates unless otherwise indicated. pIC_50_ values at the mutants were compared to wt by one-way ANOVA followed by Dunnett’s multiple comparison test (BGT1) or by multiple unpaired t-tests corrected for multiple comparisons using the Holm-Šídák method (GAT3), significance levels *p < 0.05, **p < 0.01, ***p < 0.001, ****p < 0.0001).

	IC_50_ (pIC_50_ ± S.E.M.) (µM)						
	BGT1					GAT3	
	**wt**	**Q299L**	**E52Y**	**E52A**	**E52A + Q299L**	**wt**	**L314Q**
**GABA**	**18** (4.7 ± 0.03)	**1.4** (5.9 ± 0.02)****	**1.2** (5.9 ± 0.05)****	**25** (4.6 ± 0.03)	**1.0** (6.0 ± 0.08)****	**8.2** (5.1 ± 0.02)	**5.2** (5.3 ± 0.04)*
**bicyclo-GABA**	**1.5** (5.9 ± 0.12)	**48** (4.3 ± 0.08)****	**11** (5.0 ± 0.02)****	**0.040** (7.4 ± 0.10)**	**41** (4.4 ± 0.09)****	**610** (3.2 ± 0.18)	**16** (4.9 ± 0.14)**
**2**	**9.8** (5.0 ± 0.06)	**59** (4.3 ± 0.14)**	**20** (4.7 ± 0.06)	**0.12** (6.9 ± 0.05)****	**44** (4.5 ± 0.3)^a^	**211** (3.7 ± 0.04)	**291** (3.5 ± 0.03)*

a No statistical analysis was performed due to the combination of a low expression level and limited amount of compound available, and thus only n = 2.

## Discussion

By applying molecular docking and MD simulations, we derived a binding hypothesis for bicyclo-GABA and **1**. According to this hypothesis, the carboxyl group of both compounds coordinates Na1 in BGT1, whereas the protonated nitrogen moieties form hydrogen bonds with Q299 and E52. Based on this hypothesis we identified possible sites for derivatization for both compounds. The derivatization was pursued both to increase the potential of blood-brain barrier penetration, which we expected to be low for bicyclo-GABA due to a logP of −2.5 (calculated with https://chemicalize.com/) (Pajouhesh and Lenz, 2005), and, further, to reduce any off-target effects at other GABA targets such as GABA_A_ receptors. Based on the docking and MD studies, as well as what was chemically feasible, we identified the amino group of bicyclo-GABA and carbon 2 (C2) of **1** as possible sites for derivatization. Although our homology models predicted only limited space at the amino group of bicyclo-GABA, we found this derivatization site particularly interesting because previous studies have reported steric hindrance in the proximity of the cationic moiety of GABA_A_ ligands to decrease agonistic activity (Krogsgaard-Larsen and Johnston, 1978; Haefliger et al., 1984; Falch et al., 1986; Petersen et al., 2014). Derivatization at C2 of **1** was chosen because of its proximity to the extracellular lid according to the docking studies that possibly allows bulky substituents to extend into the extracellular vestibule. Accordingly, the derivatives **2** (methyl) and **3** (dimethyl) of bicyclo-GABA and the analogs **4a** (methyl)**, 4b (**ethyl**)** and **4c** (phenyl) of **1** were synthesized. **2** and **3** were prepared *via* alkylation or reductive amination from bicyclo-GABA (Figure 3). The synthesis of **4a–c** was challenging because these compounds contain a key chiral trisubstituted cyclopropane structure, which required Pd(II)-catalyzed tertiary C(sp^3^)–H alkylation and arylation *via* directing group-mediated C–H activation.

In the initial pharmacological screening, only **2** and **4a** displayed more than 50% inhibition. This indicates that our prediction of limited space at the amino group of bicyclo-GABA was correct since only methyl (**2**) was tolerated in contrast to dimethyl (**3**). However, since the analogs **4b–c,** which are the C2-ethyl/phenyl derivatives of **1,** rendered inactive, we failed to accommodate the bulky substituents in the extracellular vestibule, as only a methyl substituent (**4a**) was tolerated at C2 of **1**. We speculate that the rather flexible scaffold of compounds **4b–c** compared to bicyclo-GABA and **2** might be hampered by the attached bulk to adopt the active *syn* conformation as we only observed the syn conformation in the docking for **4a** and **4b**, but not **4c** (Kobayashi et al., 2014). Furthermore, we have to acknowledge limited predictive power of the used BGT1 homology model in the extracellular vestibule due to a one amino acid insertion in the middle of transmembrane 10. This insertion, which is located right between the interface of the orthosteric site and the extracellular vestibule, is a unique feature of the GATs (mouse, human, rat) and it is not observed in any of the other related SLC6 (e.g., monoamine transporters) transporters or related bacterial homologs (Beuming et al., 2006; Kickinger et al., 2019; Colas, 2020). Even though an extended conformation such as a π-helix was suggested for this insertion (Dayan et al., 2017), and an extended conformation is also present in the used homology model, the correct architecture of this feature will be only known upon the release of a BGT1 3D-structure. In order to address this issue, we performed induced-fit docking to allow flexible sidechain and backbone movements. It is therefore also not surprising that our predictions were more accurate for the bicyclo-GABA analogs **2** and **3**, since the *N*-methyl/dimethyl derivatizations were located in the lower part of the pocket, which we consider more accurately represented in the homology model. Additionally, bicyclo-GABA is a rigid analog of **1** already trapped in the active *syn* conformation rendering it the more promising candidate for derivatization. We believe that the loss of activity of the dimethyl derivative **3** could be either explained by the fact that **3** cannot undergo hydrogen bonds with Q299 and E52 or because it is simply too sterically demanding to fit into the limited space.

In order to further probe the validity of the proposed binding mode for bicyclo-GABA and its most active derivative **2**, we performed site-directed mutagenesis studies. According to the docking and MD studies, hydrogen bonding between the protonated nitrogen of bicyclo-GABA and **2** with Q299 and E52 was identified as the most relevant interactions. Q299 and E52 are not conserved among the GATs. In particular, Q299 corresponds to leucine in all other GATs and E52 corresponds to tyrosine in GAT1 (Figure 5A). In line with this hypothesis, we observed decreased inhibitory activity of bicyclo-GABA at the BGT1 mutants Q299L, E52Y and E52A + Q299. Most convincingly, we were able to introduce bicyclo-GABA activity into GAT3 by introducing the reciprocal mutation corresponding to Q299 (GAT3 mutant L314Q). In addition to strongly confirming our binding hypothesis, this also shows that Q299 in BGT1 is a trigger for activity and selectivity (Table 2). A similar conclusion was drawn for the analog **2** although the mutational data was not as clear as for bicyclo-GABA since the GAT3 L314Q mutant did not show the expected increase in activity. This could be due to the fact that **2** can undergo one hydrogen bond less than bicyclo-GABA, which can also explain why **2** is less subtype selective and shows some GAT3 activity (Table 2). In a previous study, we have already identified Q299 and E52 in BGT1 as part of the molecular determinants driving activity and selectivity of a series of cyclic 2-amino-tetrahydropyrimidine/pyridine compounds (Kickinger et al., 2020). We therefore conclude that the interaction between the amino moiety of different BGT1 compound classes with Q299 and E52 is a general key feature for selective BGT1 inhibition (Jørgensen et al., 2017; Kickinger et al., 2020; Lie et al., 2020).

Interestingly, in this study we also observed drastically increased inhibitory activities for both bicyclo-GABA and **2** at the BGT1 mutant E52A, which constitutes the only “unnatural” mutation not corresponding to any residue observed in the neurotransmitter transporters (Kickinger et al., 2019). The increase is especially remarkable for bicyclo-GABA approaching a potency of 40 nM. This finding is in line with previous findings, making this a consistent phenomenon among different compound classes (Kickinger et al., 2020). Even though we do not understand the underlying nature of this phenomenon, we speculate that it could be related to the size of the residue (bulky glutamine/tyrosine versus small alanine). However, previous MD simulations of the E52A mutant bound to the GABA analog ATPCA did not reveal additional insights into this (Kickinger et al., 2020). Most likely the used homology model does not provide the necessary accuracy to draw such conclusions. Nevertheless, based on the increased activity, we propose the E52A mutant as a potential candidate for X-ray crystallography or cryo-EM experiments. A 3D structure could possibly help understand this striking phenomenon of drastically increased compound activity at E52A, and further clarify the architecture of the insertion in TM10 and its role in binding.

To further probe the potential of bicyclo-GABA and **2** as selective BGT1 tool compounds, we scrutinized their intrinsic activity as well as their selectivity among GABAergic targets. By use of the FMP assay we show that both bicyclo-GABA and **2** are non-transported BGT1 competitive inhibitors, which is supported by the Gaddum-Schild analysis. This is a truly unexpected finding due to the small size of the compounds and their structural resemblance with GABA. However, it could possibly be explained by the fairly high apparent affinity for BGT1 (*K*_*B*_ value of 470 nM for bicyclo-GABA), possibly preventing the transporter from undergoing the conformational changes necessary for translocation. We further speculate that conformational changes of GABA may occur during the translocation by BGT1 since Skovstrup *et al.,* who initially identified an extended GABA conformation as the most plausible binding orientation in GAT1 from docking studies, observed the adaptation of a “cyclic” conformation in short-term molecular dynamic simulations (Skovstrup et al., 2010, 2012). Such conformational changes are impossible for bicyclo-GABA and **2** due to their rigid bicyclic backbone. Further studies, such as long-term molecular dynamics simulations with enhanced sampling techniques of GABA, bicyclo-GABA and **2** as well as fluorescence resonance energy transfer (FRET) studies (Zhao et al., 2010, 2011), could clarify the conformational states adopted by the ligands and the protein. Furthermore, it would be of value to obtain a radioligand or labelled version of bicyclo-GABA for direct binding studies. This would also allow for studies to discern further the selectivity for other GABA targets. Although bicyclo-GABA displays some agonistic activity at α_1_β_2_γ_2_ GABA_A_ receptors in the FMP assay, it still has 13 times preference for BGT1 according to the FMP data. In contrast, **2** shows no inhibitory activity indicating that the *N-*methyl substituent effectively hinders the interaction with GABA_A_ receptors. Thus, even though **2** is slightly less active and selective than bicyclo-GABA, it constitutes a valuable tool compound as a BGT1-selective inhibitor devoid of GABA_A_ receptor agonism.

## Conclusion

In this study we have investigated and validated the binding mode of a novel class of cyclic GABA analogs, including bicyclo-GABA, the first selective BGT1 inhibitor with the highest functional activity reported to date (Kobayashi et al., 2014). With the help of docking and MD studies, we designed and synthesized five new cyclic derivatives as potential BGT1 tool compounds. By introduction of an *N*-methyl group, GABA_A_ receptor activity was successfully omitted. Using computationally guided mutagenesis studies we reveal that interactions with the residues Q299 and E52 drive activity and selectivity of bicyclo-GABA and **2**. Since these two residues were already identified as relevant for other compound classes (Jørgensen et al., 2017; Kickinger et al., 2020; Lie et al., 2020), we conclude that hydrogen bonding between the protonated nitrogen moiety of BGT1 inhibitors and Q299 and E52 is a key feature for selective BGT1 inhibition. Although the synthetic chemistry for producing bicyclo-GABA and **2** is challenging, both compounds constitute relevant tool compounds for further pharmacological investigations, potentially including conversion into (radio)labeled ligands for BGT1 to determine affinities and/or visualize regional and cellular BGT1 localization. Such studies would substantially contribute to our understanding of the pharmacological role of BGT1 in the brain and beyond.

## Methods

### Chemistry

A detailed description of the chemical procedures can be found in the Supplementary Material S1 page 9.

### Modeling

Ligand preparation was carried out with LigPrep (LigPrep, 2019) (pH 7.4 ± 1). Protein preparation was performed with PrepWiz (LigPrep, 2019-1) (pH 7.4). All compounds were docked according to an induced fit docking protocol (Sherman et al., 2006) (extended sampling) implemented in the Schrödinger Suite 2019-1 using the OPLS3e force field (Harder et al., 2016) in an outward-occluded BGT1 homology model (Damgaard et al., 2015) based on the homologous LeuT crystal structure PDB ID 2A65 (Yamashita et al., 2005). The orthosteric site was defined as all corresponding BGT1 residues that are within 4.5 Å distance to leucine in the crystal structure 2A65 (Yamashita et al., 2005) (Supplementary Table S1). For the initial docking of bicyclo-GABA and **1**, 50 poses were generated and clustered according to the volume overlap of the common scaffold (O=C([O-])CC1C([N+])C1) into 11 clusters with the Schrödinger Suite 2019-1 applying an extended sampling protocol (Supplementary Figure S2). For docking of all analogs (**2–3**, **4a–c**) 44 poses were generated and again clustered together with the poses of bicyclo-GABA and **1** into 15 clusters (Supplementary Figure S5). All poses were analyzed according to the glide gscore and glide emodel (Friesner et al., 2004) score (Supplementary Figure S1, S2, S5). The most promising poses of bicyclo-GABA and **1** were selected from the top populated cluster according to the aforementioned scores. The poses were then simulated for 100 ns for three times. MD simulations were performed with Desmond (Bowers et al., 2006) in the Schrödinger Suite 2019-1 using the OPLS3e (Harder et al., 2016) force field, SPC solvent model, POPC (300 K) as a membrane model and 0.15 M NaCl salt concentration. The system was placed in a box with periodic boundary conditions and relaxed according to the standard protocol with membrane relaxation (NVT ensemble with short time steps with Brownian dynamics at 10 K and restrained solute heavy atoms, NVT ensemble using Berendsen thermostat, NPT ensemble using Berendsen thermostat and barostat). The production run was performed for 100ns with two replicas for each compound according to the standard protocol (NPγT ensemble at 300 K, Berendsen thermostat and barostat, recording intervals of 1.2 ps for the energy and 4.8 ps for the trajectory, Supplementary Figure S3, S4).

### Pharmacology

#### Materials

Dulbecco’s modified Eagle medium (DMEM) with GlutaMAX-I, Ham’s F12 Nutrient Mix, Dulbecco’s phosphate-buffered saline (PBS), Hank’s balanced salt solution (HBSS), fetal bovine serum (FBS), penicillin–streptomycin (P/S), hygromycin B and trypsin-EDTA were purchased from Life Technologies (Paisley, United Kingdom). HEPES (4-(2-hydroxyethyl)piperazine-1-ethanesulfonic acid), poly-D-lysine (PDL), GABA, CaCl_2_ and MgCl_2_ were purchased from Sigma-Aldrich (St. Louis, MO, United States). FLIPR Membrane Potential (FMP) blue dye was purchased from Molecular Devices (Crawley, UK). Plasmocin was purchased from InvivoGen (San Diego, CA, United States) and PolyFect Transfection Reagent from Qiagen (West Sussex, United Kingdom). [2,3-^3^H(N)]GABA (specific radioactivity 35 Ci/mmol) and MicroScint™20 were purchased from PerkinElmer (Boston, MA, United States).

#### Cell Culturing and Transient Transfection

The tsA201 cell line (a transformed HEK293 cell line), the Flp-In CHO cell lines stably expressing human (h) GATs and the HEK293 cell line stably expressing human α_1_β_2_γ_2_ GABA_A_ receptors have been described previously and were cultured accordingly (Al-Khawaja et al., 2014; Falk-Petersen et al., 2020). The tsA201 cell line used for transient expression of hGATs was transfected with DNA constructs (8 μg per 10 cm plate) using 40 μL PolyFect transfection reagent according to the manufacturer’s protocol (Qiagen). All DNA constructs used have been described previously: wt hBGT1, wt GAT3 (Kvist et al., 2009), BGT1 Q299, GAT3 L314Q (Jørgensen et al., 2017), hBGT E52Y, BGT1 E52A and BGT1 E52A + Q299L (Kickinger et al., 2020).

#### [^3^H]GABA Uptake Assay

The [^3^H]GABA competition uptake assay was performed as previously described (Kvist et al., 2009; Al-Khawaja et al., 2014). Briefly, cells were seeded in white 96-well plates (White Opaque Tissue Culture (TC) plated 96-well Microplate) pre-coated with PDL. Medium was removed on the following day and cells washed with 100 µL/well assay buffer (HBSS supplemented with 20 mM HEPES, 1 mM CaCl_2_ and 1 mM MgCl_2_, pH 7.4). Then, 75 µL/well assay buffer containing 30 nM [^3^H]GABA and various concentrations of test compounds were added to the cells and incubated for 3 min at 37°C. The cells were washed 3 times with 100 µL/well ice-cold assay buffer, 150 µL/well MicroScint™20 was subsequently added before the plate was shaken for at least 1 h. The plate was counted in a Packard TopCounter microplate scintillation counter (PerkinElmer) for 3 min per well. Typical counts per minute for the different GATs were in the range of: wt hBGT1 (100–4,000), BGT1 Q299 (100–1,000), hBGT E52Y (150–500), BGT1 E52A (150–8,000), BGT1 E52A + Q299L (150–500), wt GAT3 (200–8,000), GAT3 L314Q (100–1,500), wt GAT1 (100–10,000) and wt GAT2 (150–7,500). The [^3^H]GABA uptake data for concentration-response curves was normalized to total uptake in the presence of the lowest concentration of test compound, while data for single test concentrations was normalized to total uptake. Data presented is the pooled data of at least three independent experiments with three technical replicates if not stated otherwise in figure legends. Concentration–response curves were fitted by non-linear regression to the sigmoidal concentration-response model with GraphPad Prism (version 9.0.0, GraphPad Software, San Diego, CA, United States) as described (Lie et al., 2020).

#### FMP Assay

The FMP assay performed on human α_1_β_2_γ_2_ GABA_A_ receptors in HEK293 cells was performed as described and data analyzed accordingly (Falk-Petersen et al., 2020). Briefly, transfected HEK293 cells were plated (50,000 cells/well) into clear-bottom black 96-well plates (Falcon®96-well Black Flat Bottom TC-treated Microplate) pre-coated with PDL. The FMP assay was performed the following day where the cells were washed with 100 µL/well assay buffer (HBSS supplemented with 20 mM HEPES, 2 mM CaCl_2_ and 0.5 mM MgCl_2_, pH 7.4) followed by addition of 100 µL/well FMP blue dye (0.5 mg/ml) diluted in assay buffer. The cell plates were then incubated at 37°C with 5% CO_2_ shielded from light for 30 min. Test compound solutions were prepared at 4x concentration in assay buffer and added to ligand plates pre-heated at 37°C for 10–15 min in the FLEXstation3 plate reader (Molecular Devices). After incubation, 33.3 µL test compound solution (4x) was automatically transferred to each well in the cell plate and the signal was measured for 90 s by measuring emission at 560 nm caused by excitation at 530 nm. The experiments were performed in triplicate in three independent experiments for each compound except for **2** where only the 100 µM concentration was tested in three independent experiments as **2** displayed no agonistic activity at lower concentrations. The FMP assay performed with bicyclo-GABA and **2** on BGT1 or GAT1 stably expressed in CHO cells were performed in a similar manner to the FMP assay at α_1_β_2_γ_2_ GABA_A_ receptors. The experiments were performed in triplicate in two to five independent experiments for each compound. Data obtained in the FMP assay are given as relative changes in fluorescence units (ΔRFU) by subtracting the average of the baseline fluorescence signal from the peak fluorescence signal after compound addition. Data is normalized to GABA_max_. We sometimes observed negative ΔRFU values in the presence of bicyclo-GABA without GABA EC_50_ present. This was also observed with buffer (not shown) indicating that it is not related to bicyclo-GABA. Further, this is also a typical phenomenon in the FMP assay also for compounds completely dissolved (Falk-Petersen et al., 2021). Concentration–response curves for agonists were fitted using the four-parameter concentration-response model with GraphPad Prism as described by Falk-Petersen et al. (2020). A Gaddum-Schild plot was performed according to the equation: log(dr-1) = log(B)+pA_2_, where B is the concentration of the inhibitor, pA_2_ is the negative logarithm of the dissociation constant K_B_, and the dose-ratio dr is equal to the EC′_50_/EC_50_, where EC′_50_ and EC_50_ are determined in the presence and absence of the different inhibitor concentrations, respectively. The Gaddum-Schild plot was fitted by linear regression with variable slope using GraphPad Prism.

#### Statistical Analysis

The pIC_50_ values obtained in the [^3^H]GABA competition uptake assay at the mutants were compared to the wt transporter by one-way ANOVA followed by Dunnett’s multiple comparison test (BGT1 and mutants) or multiple unpaired t-Atests corrected for multiple comparisons using the Holm-Šídák method (GAT3 and mutants) using GraphPad Prism, as further specified in the respective figure legends.

## Data Availability

The modeling data presented in this study can be found in online repositories https://zenodo.org/ under the url https://doi.org/10.5281/zenodo.5357152. The pharmacological data is available from the corresponding authors upon request.
