# Supplementary material for: Molecular Determinants and Pharmacological Analysis for a Class of Competitive Non-transported Bicyclic Inhibitors of the Betaine/GABA Transporter BGT1
Source: Front Chem. 2021 Sep 14;9:736457. doi: 10.3389/fchem.2021.736457 (PMC8476755; doi:10.3389/fchem.2021.736457)
Supplement: Supplementary file 1 [file DataSheet1.pdf]

Supplementary Material

Table of content

Table S1. Residues of the orthosteric site of all GATs. 2

Figure S1. Distribution of glide gscore/emodel among all docking poses per compound.......... 2

Figure S2. Distribution of poses of bicyclo-GABA and **1** per cluster. 3

Figure S3. Root-mean-square-deviation and Protein-ligand-interaction schematic overview of the all MD simulations of bicyclo-GABA. 4

Figure S4. Root-mean-square-deviation and Protein-ligand-interaction schematic overview of all MD simulations of **1**.......... 5

Figure S5. Distribution of poses of bicyclo-GABA, **1-3** and **4a-c** per cluster. 6

Figure S6. Protein-ligand-interaction-fingerprint (PLIF) diagram of the most populated docking cluster 5. 7

Figure S7 Pooled and normalized response (% of $GABA_{max}$) of bicyclo-GABA at GAT1. 7

General synthetic procedures.......... 8

Figure S8. Synthesis of the trisubstituted chiral cyclopropanes **5a–c**. 9

References 12

Supplementary Table S1. Residues of the orthosteric site of all GATs. The pocket was defined as all corresponding GAT residues that are within a 4.5 Å distance to leucine in the crystal structure 2A65 (Yamashita et al., 2005). The alignment was taken from Kickinger *et al.* (Kickinger et al., 2019).

**GAT1**	Y60	A61	I62	G63	L64	G65	N66	L136	Y140	F294	S295	Y296	G297	L300	N327	S396	T400
**BGT1**	E52	I53	I54	G55	L56	G57	N58	L129	Y133	F293	S294	F295	A296	Q299	N326	S395	C399
**GAT2**	E48	I49	I50	G51	L52	G53	N54	L125	Y129	F288	S289	F290	A291	L294	N321	S390	C394
**GAT3**	E66	I67	I68	G69	L70	G71	N72	L143	Y147	F308	S309	Y310	A311	L314	N341	S410	C414

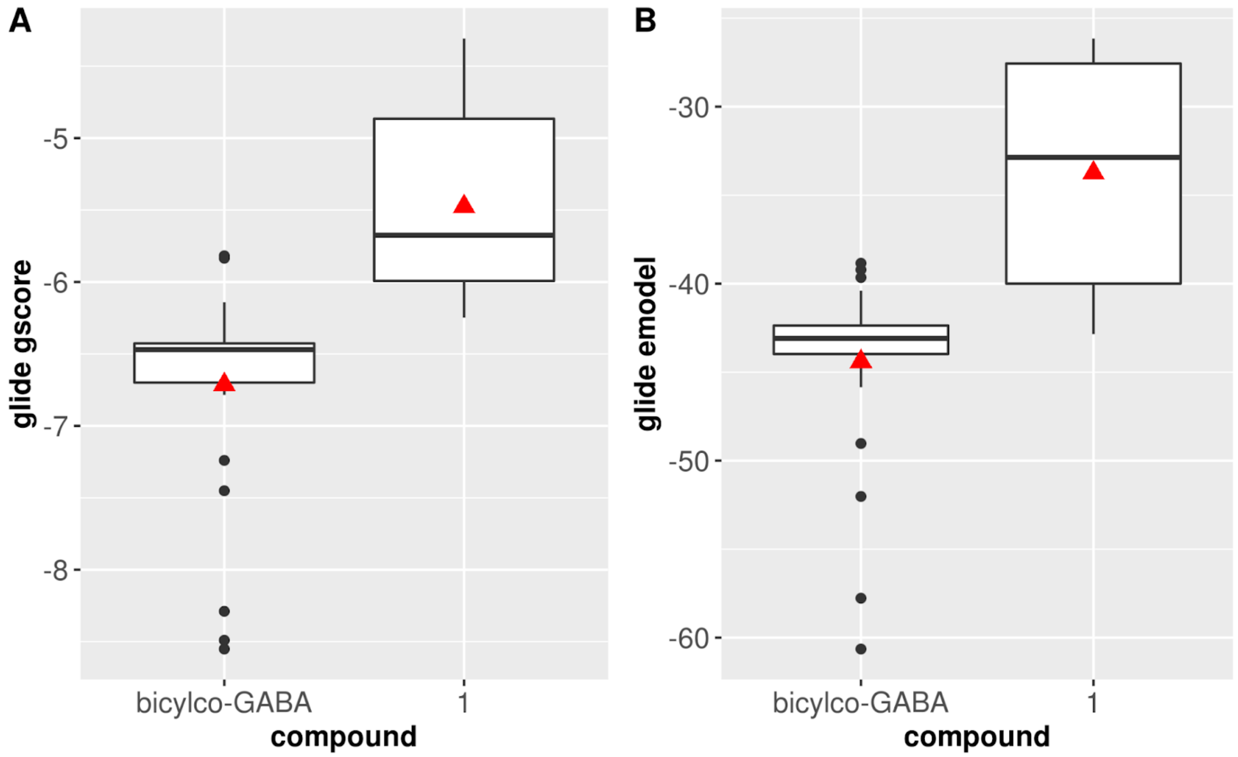

Figure S1. (**A**) Distribution of glide gscore among all docking poses per compound. The boxplots show the median as a bold line and the mean as a red triangle. The upper hinge represents the 75th percentile and the lower hinge represents the 25th percentile. The whiskers represent the 1.5 inter quartile range. (**B**) Distribution of glide emodel score among all docking poses per compound.

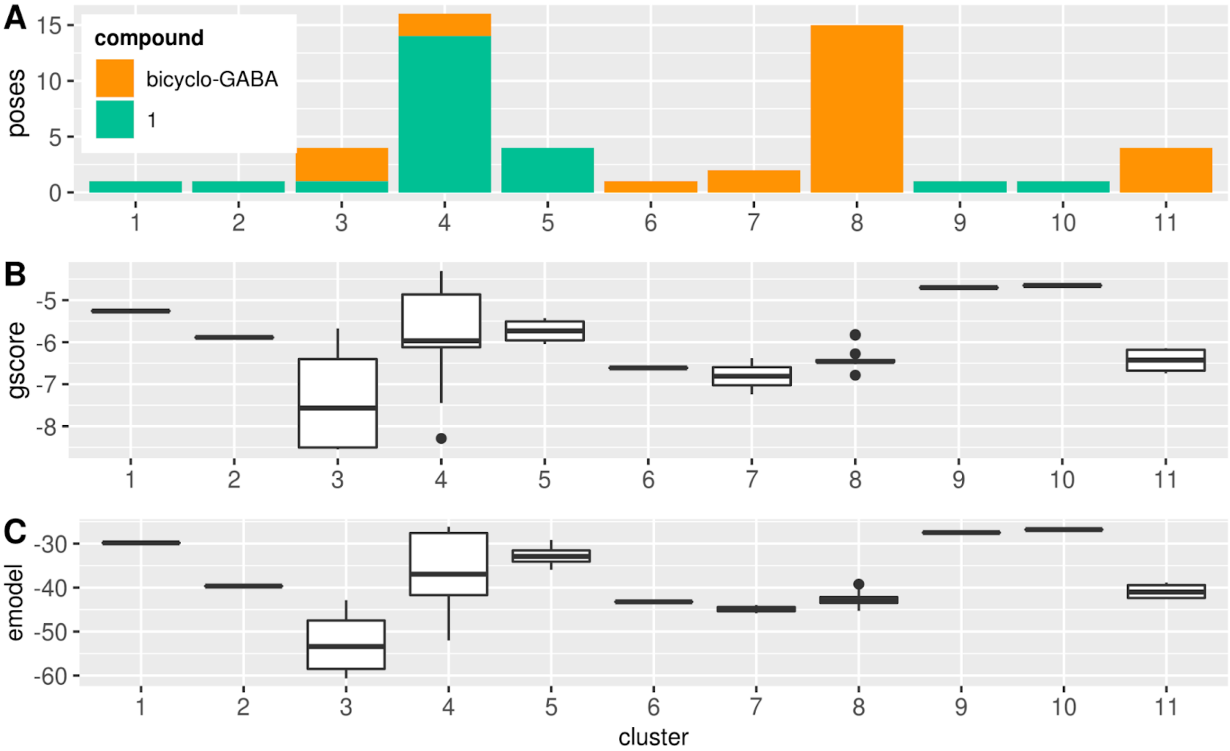

Supplementary Figure S2. **(A)** Distribution of poses of bicyclo-GABA and **1** per cluster. (**B**) Distribution of glide score per cluster. The boxplots show the median as a bold line. The upper hinge represents the 75th percentile and the lower hinge represents the 25th percentile. The whiskers represent the 1.5 inter quartile range. (**C**) Distribution of glide emodel score per cluster.

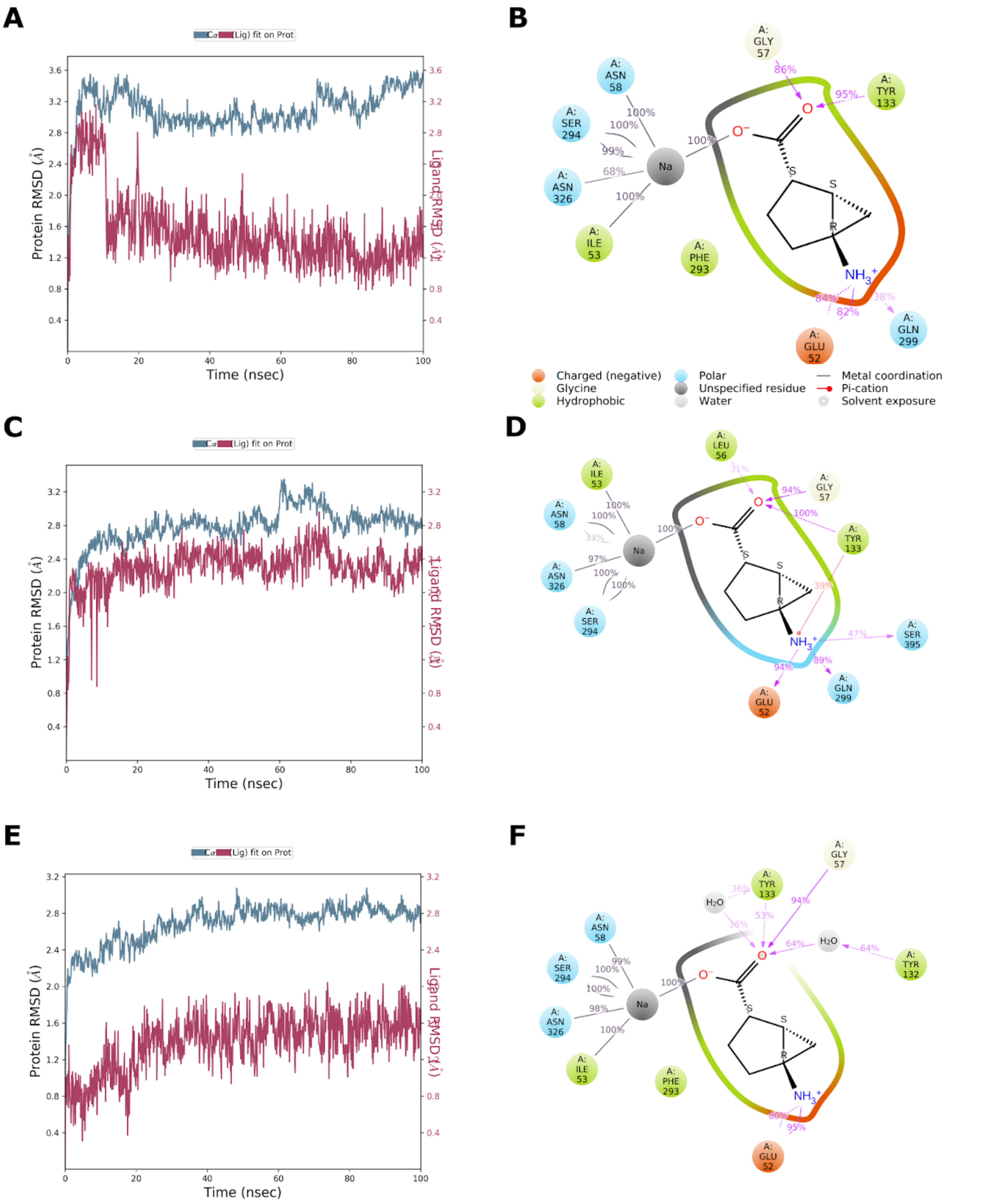

Supplementary Figure S3. (A, C, E) Root-mean-square-deviation (RMSD) of three different runs of 100 ns MD simulations of the most promising bicyclo-GABA docking pose in BGT1. (**B, D, F**) Protein-ligand-interaction schematic overview of the same MD simulations as in A, C and E.

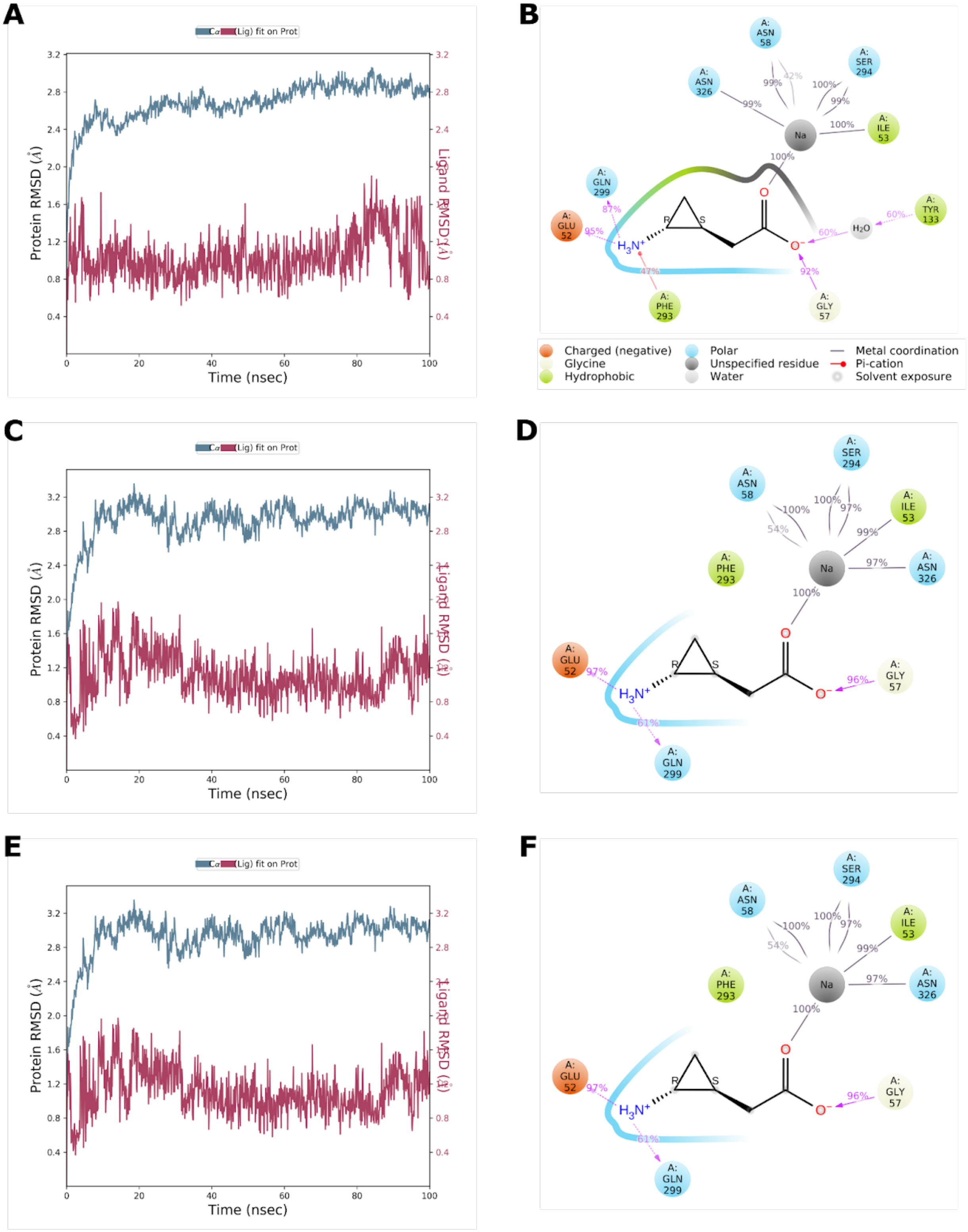

Supplementary Figure S4. Figure S3. (A, C, E) Root-mean-square-deviation (RMSD) of three different runs of 100 ns MD simulations of the most promising **1** docking pose in BGT1. (**B, D, F**) Protein-ligand-interaction schematic overview of the same MD simulations as in A, C and E.

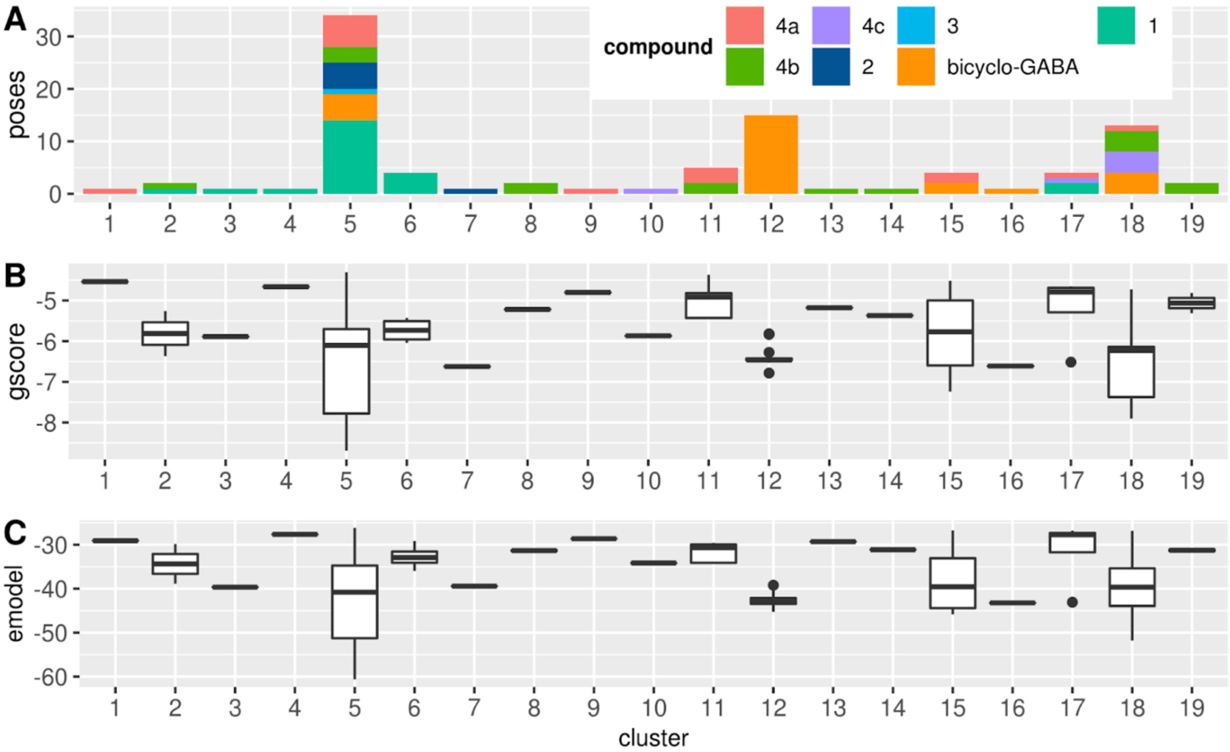

Supplementary Figure S5. A) Distribution of poses of bicyclo-GABA, **1-3** and **4a-c** per cluster. The boxplots show the median as a bold line. The upper hinge represents the 75th percentile and the lower hinge represents the 25th percentile. (**B**) Distribution of glide score per cluster. (**C**) Distribution of glide emodel score per cluster.

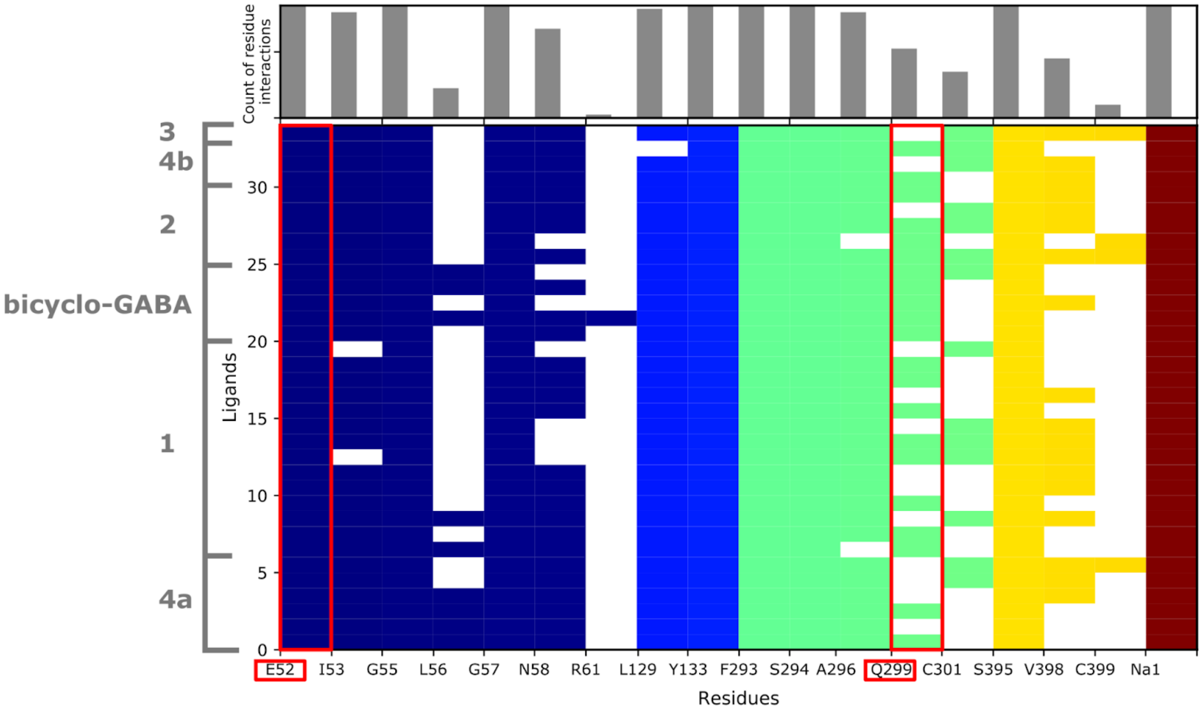

Figure S6. Protein-ligand-interaction-fingerprint (PLIF) diagram of the most populated docking cluster 5. The PLIFs were generated with the Schrodinger Suit 2019-1 with default settings. The color coding is according to the location of the residues within the different transmembrane helices (dark blue helix 1, blue helix 3, green helix 6, yellow helix 8, brown metal coordination).

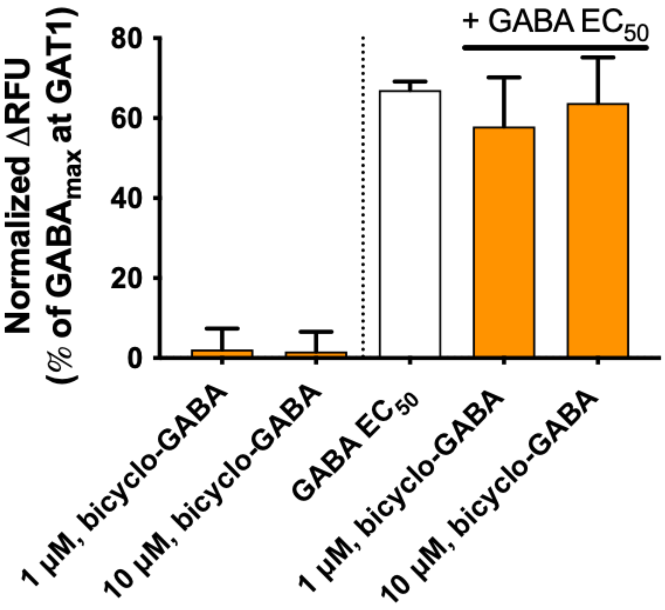

Figure S7. Pooled and normalized response (% of $GABA_{max}$) of bicyclo-GABA without and with GABA EC_{50} (10 µM GABA) present at GAT1 (n=2). Data were compared to GABA EC_{50} by a One-

way ANOVA followed by Dunnett's multiple comparison test, significance levels P>0.05. Data are presented as means ± S.E.M.

General synthetic procedure

All ^{1}H NMR and ^{13}C NMR spectra were recorded on a JEOL JNM-AL-400, JEOL JMM-ECX-400P or JEOL JMM-ECA-500 spectrometer. ^{1}H NMR chemical shifts are reported as d values in ppm relative to tetramethylsilane (0.00 ppm) when $CDCl_3$ was used as the solvent, or a solvent residual peak ($CD_2\underline{H}$-OD: 3.31 ppm) when CD_3OD was used as the solvent. Coupling constants (*J*) are reported in Hz and multiplicity is indicated as follows: s (singlet), d (doublet), dd (double doublet), t (triplet), q (quartet), brs (broad singlet). ^{13}C NMR chemical shifts are reported as d values in ppm relative to deuterated solvent ($\underline{C}DCl_3$: 77.0 ppm or $\underline{C}D_3OD$: 49.0 ppm). All mass spectra were obtained on a JEOL JMS-700TZ, JMS-HX11 or JEOL JMS-FABmate spectrometer. Elemental analysis was performed with a Yanaco CHN Corder MT-6 or J-Science MICRO CODER JM10 analyzer. Optical rotations were measured with a JASCO P-1030 digital polarimeter. All non-aqueous reactions were carried out under argon atmosphere in anhydrous grade solvents. Silica gel column chromatography was performed using Merck Silica Gel 60 or Kanto Silica Gel 60. TLC was performed using glass-backed silica gel 60F254.

Compound 2. A mixture of bicyclo-GABA (HCl salt, 18 mg, 0.10 mmol), Boc_2O (33 mg, 0.15 mmol), and Et_3N (21 mL, 0.15 mmol) in MeOH (1 mL) was stirred at room temperature for 10 h (Figure 3). The solvent was evaporated and the residue was dissolved in DMF (1.0 mL). To the solution were added CH_3I (19 µL, 0.30 mmol) and NaH (60% dispersion in paraffin liquid, 12 mg, 0.28 mmol) at 0 °C, and the mixture was stirred at room temperature for 24 h. The resulting mixture was partitioned between $CHCl_3$ and aq. HCl (1 M). The organic layer was dried (Na_2SO_4) and evaporated. The residue was passed through a silica gel pad (hexane/AcOEt = 1:1) and the filtrate was evaporated. The resulting residue was dissolved in HCl solution (4 M in dioxane, 1.0 mL), and the mixture was stirred at room temperature for 1 h. After removal of the solvent *in vacuo*, the residue was triturated with dry Et_2O. The precipitation was filtered off and dried to give **2** (HCl salt, 17 mg, 86 µmol, 86%) as a white powder. $[\alpha]_D^{16} = -64.2°$ (*c* 0.65, CH_3OH); ^{1}H NMR (500 MHz, CD_3OD) δ 3.23 (1 H, m), 2.75 (3 H, s), 2.23 (1 H, m), 2.16–1.98 (3 H, m), 1.69 (1 H, m), 1.15–1.13 (2 H, m); ^{13}C NMR (125 MHz, CD_3OD) δ 176.7, 48.7, 44.4, 31.8, 28.3, 25.9, 24.8, 10.5; LRMS (ESI) *m/z* 156 [$(M + H)^+$]; Anal. calcd for $C_5H_9NO_3$•1HCl•0.1H_2O: C, 49.67; H, 7.40; N, 7.24. Found: C, 49.40; H, 7.31; N, 7.10.

Compound 3. To a solution of bicyclo-GABA (HCl salt, 12 mg, 0.068 mmol) and aq. HCHO (36%, 13 µL, 0.17 mmol) in MeOH (1.0 mL) was added $NaBH_3CN$ (13 mg, 0.20 mmol), and the mixture was stirred at room temperature for 7 h (Figure 1). After removal of the solvent *in vacuo*, the residue was passed through a silica gel pad ($CHCl_3$/MeOH = 4:1–1:1) and the filtrate was evaporated. The resulting residue was dissolved in HCl solution (4 M in dioxane, 1.0 mL) and the mixture was stirred at room temperature for 1.5 h. After removal of the solvent *in vacuo*, the residue was triturated with dry Et_2O. The precipitation was filtered off and dried to give **3** (HCl salt, 12 mg, 60 µmol, 88%) as a white powder. $[\alpha]_D^{16} = -44.3°$ (*c* 0.37, CH_3OH); ^{1}H NMR (500 MHz, CD_3OD) δ 3.29 (1 H, m), 2.95 (6 H, s), 2.30–2.13 (3 H, m), 2.03 (1 H, m),1.59 (1 H, m), 1.32 (1 H, m), 1.22 (1 H, m); ^{13}C NMR (125 MHz, CD_3OD) δ 176.5, 55.7, 44.3, 41.7, 27.0, 24.8, 24.4, 12.1; LRMS (ESI) *m/z* 170 [$(M + H)^+$]; Anal. calcd for $C_5H_9NO_3$•2HCl: C, 44.64; H, 7.08; N, 5.78. Found: C, 44.24; H, 6.69; N, 5.82.

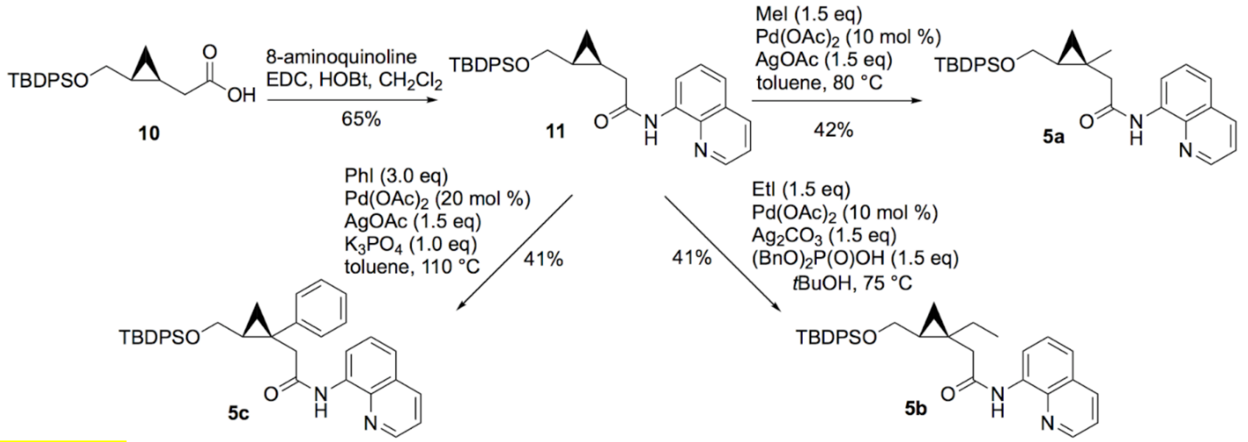

Figure S8. Synthesis of the trisubstituted chiral cyclopropanes **5a–c**.

Compound 11. To a solution of carboxylic acid **10** (Suemasa et al., 2018) (3.68 g, 10.0 mmol) in CH_2Cl_2 (100 mL) were added EDC (2.30 g, 12.0 mmol) and HOBt (1.35 g, 10.0 mmol) at 0 °C and the mixture was stirred at 0 °C. After 15 min, 8-aminoquinoline (1.73 g, 12.0 mmol) was added to the mixture and the reaction mixture was stirred at room temperature for 22 h (Figure S8). The resulting mixture was diluted with $CHCl_3$, washed with aq. HCl (1 M), saturated aq. $NaHCO_3$ and H_2O, dried (Na_2SO_4), and evaporated. The residue was purified by silica gel column chromatography (hexane/AcOEt = 6:1) to give **11** (3.19 g, 64.5 mmol, 65%) as a pale yellow powder. ^{1}H NMR (500 MHz, $CDCl_3$): δ 10.2 (1 H, s, NH), 8.77 (1 H, m, aromatic), 8.54 (1 H, m, aromatic), 8.09 (1 H, m), 7.68–7.66 (4 H, m), 7.54–7.47 (2 H, m), 7.40–7.31 (7 H, m), 3.74–3.71 (2 H, m,), 3.69 (1 H, dd, J = 10.5, 4.7 Hz), 2.52 (1 H, dd, J = 16.0, 6.0 H), 2.45 (1 H, dd, J = 16.0, 6.7 Hz), 1.12 (1 H, m), 1.03 (9 H, s), 0.75 (1 H, dd, J = 12.5, 5.5 Hz), 0.55 (1 H, dd, J = 12.5, 5.2 Hz). The ^{1}H NMR data were in agreement with those of its enantiomer reported previously (Hoshiya et al., 2013).

Compound 5a. The mixture of **11** (494 mg, 1.0 mmol), CH_3I (93 mL, 1.5 mmol), AgOAc (250 mg, 1.5 mmol), and $Pd(OAc)_2$ (23 mg, 10 mol %) in dry toluene (5.0 mL) was stirred at 80 °C for 24 h (Figure S8). The reaction mixture was cooled to room temperature and filtered through a Celite pad. The filtrate was concentrated *in vacuo* to remove toluene. The residue was purified by flash silica gel column chromatography (hexane/AcOEt = 30:1) to give **5a** (212 mg, 42%) as a colorless oil. ^{1}H NMR (400 MHz, $CDCl_3$) δ 10.2 (1 H, s), 8.79 (1 H), 8.61 (1 H), 8.13 (1 H), 7.70–7.67 (4 H), 7.55–7.49 (2 H), 7.43–7.26 (7 H), 3.90–3.82 (2 H), 2.52 (1 H, d, J = 15.6 Hz), 2.42 (1 H, d, J = 15.6 Hz), 1.28–1.21 (4 H, m), 1.01 (9 H, s), 0.86 (1 H, m), 0.45 (1 H, m); ^{13}C NMR (100 MHz, $CDCl_3$) δ 170.4, 148.2, 138.5, 136.2, 135.6, 134.6, 134.0, 133.9, 129.5, 127.9, 127.6, 127.6, 127.4, 121.5, 121.3, 116.4, 64.1, 49.7, 26.8, 25.7, 19.1, 18.0, 17.7, 17.4; The ^{1}H NMR data were in agreement with those of its enantiomer reported previously (Hoshiya et al., 2016).

Compound 5b. The mixture of **11** (1.48 g, 3.00 mmol), ethyl iodide (720 μL, 9.00 mmol), Ag_2CO_3 (1.08 g, 3.90 mmol), $(BnO)_2PO_2H$ (1.08 g, 3.90 mmol), and $Pd(OAc)_2$ (135 mg, 20 mol %) in dry *t*BuOH (6.0 mL) was stirred at 75 °C for 36 hr (Figure S8). The reaction mixture was cooled to room temperature and filtered through a Celite pad. The filtrate was concentrated *in vacuo* to remove *t*BuOH. The residue was purified by flash silica gel column chromatography (hexane/AcOEt = 30:1) to give **5b** (634 mg, 41%) as a colorless oil. ^{1}H NMR (500 MHz, $CDCl_3$) δ 10.2 (1 H, s), 8.77 (1 H, m), 8.58 (1 H, m), 8.10 (1 H, m), 7.69–7.67 (4 H, m), 7.53-7.46 (2 H, m), 7.40–7.33 (7 H, m), 3.95 (1 H, dd, J = 11.0, 7.0 Hz), 3.80 (1 H, dd, J = 11.0, 7.0 Hz), 2.54 (1 H, d, J = 15.5 Hz), 2.47 (1 H, d, J = 15.5 Hz), 1.56–1.51 (2 H, m) 1.28 (1 H, m), 1.14 (3 H, d, J = 7.5 Hz), 1.01 (9 H, s), 0.84 (1 H, m), 0.42 (1 H, m).

The ^{1}H NMR data were in agreement with those of its enantiomer reported previously (Hoshiya et al., 2016).

Compound 5c. The mixture of **11** (25 mg, 50 µmol), iodobenzene (16 mL, 150 µmol), AgOAc (13 mg, 75 mmol), K_3PO_4 (11 mg, 50 mmol), and $Pd(OAc)_2$ (2.2 mg, 20 mol%) in dry toluene (250 µL) was stirred at 110 °C for 24 h (Figure S8). The mixture was cooled to room temperature and filtered through a Celite pad. The filtrate was concentrated *in vacuo* to remove toluene. The residue was purified by flash silica gel column chromatography (hexane/AcOEt = 10:1) to give **5c** (12 mg, 21 µmol, 41%) as a colorless oil. ^{1}H NMR (500 MHz, $CDCl_3$) δ 9.97 (1 H, s), 8.70–8.69 (2 H, m), 8.09 (1 H, m), 7.63–7.15 (18 H, m), 3.51 (1 H, dd, J = 11.0, 6.0 Hz), 3.35 (1 H, d, J = 14.5 Hz), 3.15 (1 H, dd, J = 11.0, 6.0 Hz), 2.47 (1 H, d, J = 14.5 Hz), 1.57 (1 H, m), 1.21 (1 H, m), 1.11 (1 H, m), 0.99 (9 H, s). The ^{1}H NMR data were in agreement with those of its enantiomer reported previously (Hoshiya et al., 2013).

Compound 6a. A mixture of **5a** (200 mg, 0.39 mmol), DMAP (96 mg, 0.76 mmol) and Boc_2O (270 µL, 1.2 mmol) in CH_3CN (4.0 mL) was stirred at 70 °C for 4 h. The mixture was concentrated *in vacuo* and passed through a silica gel pad (hexane/AcOEt = 5:1), and the filtrate was evaporated (Figure 4). To a solution of the residue in aq. THF (80%, 4.0 mL) were added aq. H_2O_2 (30 wt%, 220 mL, 23 mmol) and aq. $LiOH{\cdot}H_2O$ (1 M, 9.1 mL, 9.1 mmol) at 0 °C, and the mixture was stirred at room temperature for 5 h. After addition of saturated aq. Na_2SO_3, the resulting mixture was extracted with CH_2Cl_2. The organic layer was washed with aq. HCl (1 M), H_2O and brine, dried (Na_2SO_4), and evaporated. The residue was purified by flash silica gel column chromatography (hexane/AcOEt = 6:1–3:1) to give **6a** (141 mg, 0.37 mmol, 93%) as a colorless oil. $[\alpha]_D^{17}$ = +7.03° (c 1.14, $CHCl_3$); ^{1}H NMR (400 MHz, $CDCl_3$) δ 7.70–7.66 (4 H, m), 7.42–7.36 (6 H, m), 3.83 (1 H, dd, J = 11.2, 5.6 Hz), 3.51 (1 H, dd, J = 11.2, 8.8 Hz), 2.29 (1 H, d, J = 15.6 Hz), 2.22 (1 H, d, J = 15.6 Hz), 1.17 (3 H, s), 1.11–1.05 (10 H, m), 0.64 (1 H, m), 0.21 (1 H, m); ^{13}C NMR (100 MHz, $CDCl_3$) δ 177.8, 135.6, 133.8, 129.6, 127.6, 64.3, 45.5, 26.8, 25.6, 19.1, 17.6, 17.2, 17.1; HRMS (ESI) calcd for $C_{23}H_{30}O_3SiNa$: 405.1862, found 405.1857 $[(M + Na)^+]$.

Compound 6b. Compound **6b** (44 mg, 0.11 mmol, colorless oil, 95%) was obtained from **5b** (60 mg, 0.13 mmol) as described for the preparation of **6a (**Figure 4**)**. $[\alpha]_D^{17}$ = +5.25° (c 1.38, $CHCl_3$); ^{1}H NMR (400 MHz, $CDCl_3$) δ 7.70–7.66 (4 H, m), 7.42–7.36 (6 H, m), 3.80 (1 H, dd, J = 11.6, 6.0 Hz), 3.55 (1 H, dd, J = 11.6, 6.8 Hz), 2.37 (1 H, d, J = 15.6 Hz), 2.21 (1 H, d, J = 15.6 Hz), 1.51 (1 H, m), 1.40 (1 H, m), 1.17 (3 H, s), 1.16–0.98 (13 H, m, H-2), 0.62 (1 H, m), 0.19 (1 H, m); ^{13}C NMR (100 MHz, $CDCl_3$) δ 177.6, 135.6, 133.7, 129.6, 127.6, 63.9, 41.7, 26.8, 26.4, 23.8, 22.6, 19.1, 16.2, 11.1; HRMS (ESI) calcd for $C_{24}H_{32}O_3SiNa$: 419.2018, found 419.2013 $[(M + Na)^+]$, calcd for $C_{24}H_{31}O_3Si$: 395.2042, found 395.2119 $[(M - H)^-]$.

Compound 6c. Compound **6c** (63 mg, 0.14 mmol, colorless oil, 94%) was obtained from **5c** (86 mg, 0.15 mmol) as described for the preparation of **6a (**Figure 4**)**. $[\alpha]_D^{17}$ = −35.1° (c 0.94, $CHCl_3$); ^{1}H NMR (500 MHz, $CDCl_3$) δ 7.62–7.61 (2 H), 7.41–7.23 (13 H, m), 3.47 (1 H, dd, J= 11.0, 5.0 Hz), 3.05 (1 H, dd, J = 11.0, 8.5 Hz), 2.95 (1 H, d, J = 15.0 Hz), 2.29 (1 H, d, J = 15.0 Hz), 1.41 (1 H, m), 1.00 (9 H, s), 0.97 (1 H, m), 0.84 (1 H, m); ^{13}C NMR (125 MHz, $CDCl_3$) δ 177.0, 139.7, 135.5, 135.4, 133.8, 133.6, 130.4, 129.5, 129.4, 128.1, 127.6, 127.5, 126.7, 64.3, 46.0, 27.8, 26.8, 26.7, 19.1, 14.5; HRMS (ESI) calcd for $C_{28}H_{32}O_3SiNa$: 467.2018, found 467.2004 $[(M + Na)^+]$, calcd for $C_{28}H_{31}O_3Si$: 443.2042, found 443.2096 $[(M - H)^-]$.

Compound 7a. A mixture of **6a** (189 mg, 0.49 mmol), K_2CO_3 (102 mg, 0.74 mmol) and BnBr (90 µL, 0.74 mmol) in DMF (5.0 mL) was stirred at 0 °C for 5 h (Figure 4). The resulting mixture was partitioned between CH_2Cl_2 and H_2O, and the organic layer was dried (Na_2SO_4) and evaporated. The residue was passed through a silica gel pad (hexane/AcOEt = 8:1), and the filtrate was evaporated. To

a solution of the residue in THF (5.0 mL) were added AcOH (70 μL, 1.3 mmol) and TBAF (1 M soln. in THF, 750 μL, 0.75 mmol), and the mixture was stirred at room temperature for 36 h. After removal of THF *in vacuo*, the residue was purified by silica column chromatography (hexane/AcOEt = 8:1–1:1) to give **7a** (94 mg, 0.40 mmol, 81%) as a colorless oil. $[\alpha]_D^{14}$ = +37.4° (*c* 0.44, $CHCl_3$); ^1H NMR (500 MHz, $CDCl_3$) δ 7.39–7.33 (5 H, m), 5.14 (2 H, d, *J* = 4.0 Hz), 3.83 (1 H, dd, *J* = 10.5, 6.0 Hz), 3.36 (1 H, dd, *J* = 10.5, 10.5 Hz), 2.59 (1 H, d, *J* = 16.0 Hz), 2.02 (1 H, d, *J* = 16.0 Hz), 2.01 (1 H, s), 1.18 (3 H, s), 1.07 (1 H, m), 0.67 (1 H, m), 0.29 (1 H, m); ^{13}C NMR (100 MHz, $CDCl_3$) δ 172.7, 135.8, 134.8, 128.6, 128.4, 66.4, 63.3, 44.8, 25.8, 17.6, 17.4, 16.8; HRMS (ESI) calcd for $C_{14}H_{18}O_3Na$: 257.1154, found 257.1157 $[(M + Na)^+]$.

Compound 7b. Compound **7b** (140 mg, 0.56 mmol, colorless oil, 97%) was obtained from **6b** (230 mg, 0.58 mmol) as described for the preparation of **7a** (Figure 4). $[\alpha]_D^{24}$ = +45.8° (*c* 0.17, $CHCl_3$); ^1H NMR (500 MHz, $CDCl_3$) δ 7.40–7.31 (5 H, m), 5.13 (2 H, d, *J* = 6.5 Hz), 3.83 (1 H, dd, *J* = 10.5, 5.5 Hz), 3.42 (1 H, dd, *J* = 10.5, 10.5 Hz), 2.69 (1 H, d, *J* = 17.0 Hz), 2.01 (1 H, s), 1.93 (1 H, d, *J* = 17.0 Hz), 1.63 (1 H, m), 1.28 (1 H, m), 1.11 (1 H, m), 0.95 (3 H, dd, *J* = 10.0, 10.0 Hz), 0.65 (1 H, m), 0.27 (1 H, m); ^{13}C NMR (100 MHz, $CDCl_3$) δ 172.7, 135.8, 134.8, 129.4, 128.6, 128.3, 127.6, 66.3, 62.7, 41.0, 26.7, 23.4, 22.4, 16.1, 11.0; HRMS (ESI) calcd for $C_{15}H_{20}O_3Na$: 271.1310, found 271.1315 $[(M + Na)^+]$.

Compound 7c. Compound **7c** (70 mg, 0.26 mmol, colorless oil, 88%) was obtained from **6c** (114 mg, 0.30 mmol) as described for the preparation of alcohol **7a** (Figure 4). $[\alpha]_D^{26}$ = −30.6° (*c* 1.03, $CHCl_3$); ^1H NMR (400 MHz, $CDCl_3$) δ 7.32–7.17 (10 H, m), 5.00 (2 H, s), 3.32 (1 H, dd, *J* = 11.2, 5.6 Hz), 3.18 (1 H, dd, *J* = 11.2, 7.6 Hz), 2.90 (1 H, d, *J* = 15.6 Hz), 2.39 (1 H, d, *J* = 15.6 Hz), 1.67 (1 H, s), 1.44 (1 H, m), 1.08 (1 H, m), 0.99 (1 H, m); ^{13}C NMR (100 MHz, $CDCl_3$) δ 171.4, 139.9, 135.7, 129.8, 128.4, 128.1, 128.1, 126.9, 66.1, 63.6, 46.1, 27.9, 26.5, 15.1; HRMS (ESI) calcd for $C_{19}H_{20}O_3Na$: 319.1310, found 319.1315 $[(M + Na)^+]$.

Compound 9a. To a solution of **7a** (300 mg, 1.28 mmol) in CH_2Cl_2 (13 mL) was added Dess–Martin periodinane (1.36 g, 3.21 mmol) at 0 °C, and the mixture was stirred at room temperature for 3 h (Figure 4). After addition of a mixture of saturated aq. $Na_2S_2O_3$ and saturated aq. $NaHCO_3$ (1:1), the resulting mixture was stirred vigorously and then extracted with $CHCl_3$. The organic layer was washed with H_2O, dried (Na_2SO_4), and evaporated to give a colorless oil. To a suspension of the oil in aq. *t*BuOH (80%, 15 mL) were added 2-methyl-2-butene (1.10 mL, 10 mmol), NaH_2PO_4 (399 mg, 2.6 mmol) and $NaClO_2$ (463 mg, 5.2 mmol) at 0 °C, and the mixture was stirred at room temperature for 5 h. The resulting mixture was partitioned between $CHCl_3$ and aq. HCl (1 M), and the organic layer was washed with brine, dried (Na_2SO_4), and evaporated. The residue was passed through a silica gel pad (hexane/AcOEt = 4/1), and the filtrate was evaporated to give **8a** as colorless oil. To a solution of the obtained **8a** in CH_2Cl_2 (12 mL) were added Et_3N (1.8 mL, 13 mmol) and DPPA (820 μL, 3.9 mmol), and the mixture was stirred at room temperature for 5 h. The mixture was partitioned between AcOEt and H_2O, and the organic layer was washed with brine, dried (Na_2SO_4), and evaporated. The residue was passed through a silica gel pad (hexane/AcOEt = 20/1) to give a colorless oil. A solution of the oil in *t*BuOH (20 mL) was stirred under reflux conditions for 19 h. After removal of the solvent *in vacuo*, the residue was purified by silica gel column chromatography (hexane/AcOEt = 8/1) to give **9a** (189 mg, 0.52 mmol, white solid,46%). $[\alpha]_D^{17}$ = −9.36° (*c* 0.25, $CHCl_3$); m.p. 71.5–72.5 °C; ^1H NMR (500 MHz, $CDCl_3$) δ 7.36–7.30 (5 H, m), 5.13 (2 H, s), 4.70 (1 H, s), 2.49 (1 H, d, *J* = 14.5 Hz), 2.43 (1 H, brs), 2.12 (1 H, d, *J* = 14.5 Hz), 1.44 (9 H, s), 1.15 (3 H, s), 0.93 (1 H, m), 0.43 (1 H, brs); ^{13}C NMR (125 MHz, $CDCl_3$) δ 171.7, 156.6, 136.0, 128.5, 128.3, 128.1, 79.3, 66.1, 43.3, 33.9, 28.3, 19.2, 18.9, 17.0; HRMS (ESI) calcd for $C_{18}H_{25}NO_4Na$: 342.1681, found 342.1682 $[(M + Na)^+]$.

Compound 9b. Compound **9b** (76 mg, 0.23 mmol, white solid, 42%) was obtained from **7b** (135 mg, 0.54 mmol) as described for the preparation of **9a** (Figure 4). $[\alpha]_D^{18}$ = −7.15° (*c* 0.32, $CHCl_3$); m.p. 98.0–98.5 °C; ^1H NMR (500 MHz, $CDCl_3$) δ 7.36–7.31 (5 H, m), 5.12 (2 H, s), 4.65 (1 H, s), 2.52 (1

H, brs), 2.44 (1 H, d, J = 154.5 Hz), 2.18 (1 H, d, J = 14.5 Hz), 1.49–1.44 (11 H, m), 0.97–0.92 (4 H, m), 0.42 (1 H, brs); ^{13}C NMR (125 MHz, $CDCl_3$) δ 171.8, 156.6, 136.0, 128.5, 128.2, 79.4, 66.2, 39.7, 34.5, 28.3, 23.8, 23.6, 18.4, 10.7; HRMS (ESI) calcd for $C_{19}H_{27}NO_4Na$: 356.1838, found 356.1838 [(M + Na)$^+$].

Compound 9c. Compound **9c** (49 mg, 0.13 mmol, white solid, 42%) was obtained from **7c** (91 mg, 0.31 mmol) as described for the preparation of **9a** (Figure 4). $[\alpha]_D^{17}$ = +43.3° (c 0.20, $CHCl_3$); m.p. 81.0–81.5 °C; ^{1}H NMR (500 MHz, $CDCl_3$) δ 7.31–7.17 (10 H, m), 5.00 (2 H, s), 4.18 (1 H, s), 3.07 (1 H, d, J = 14.5 Hz), 2.91 (1 H, brs), 2.23 (1 H, d, J = 14.5 Hz), 1.47 (1 H, brs), 1.35 (9 H, s), 1.13 (1 H, m); ^{13}C NMR (100 MHz, $CDCl_3$) δ 171.0, 156.2, 138.5, 135.8, 130.2, 128.5, 128.4, 128.1, 128.1, 127.1, 79.4, 66.1, 43.5, 34.4, 28.3, 28.2, 17.8; HRMS (ESI) calcd for $C_{23}H_{27}NO_4Na$: 404.1838, found 404.1837 [(M + Na)$^+$].

Compound 4a. A solution of **9a** (16 mg, 50 μmol) in aq. HCl (4 M, 1.0 mL) was stirred under reflux conditions for 8 h (Figure 4). After removal of the solvent *in vacuo*, the residue was partitioned between H_2O and $CHCl_3$. The aqueous layer was evaporated to give **4a** (9 mg, 50 μmol, quant.) as a white solid. $[\alpha]_D^{16}$ = +36.1° (c 0.22, CH_3OH); ^{1}H NMR (500 MHz, CD_3OD) δ 2.58 (1 H, dd, J = 4.5, 7.5 Hz), 2.40 (1 H, d, J= 16.5 Hz), 2.24 (1 H, d, J = 16.5 Hz), 1.30 (3 H, s), 1.02 (1 H, t, J = 7.5 Hz), 0.75 (1 H, dd, J = 4.5, 7.5 Hz); ^{13}C NMR (125 MHz, CD_3OD) δ 174.9, 43.2, 33.7, 18.1, 17.8, 16.7; LRMS (ESI) m/z 130 [(M + H)$^+$]; Anal. calcd for $C_6H_{11}NO_2$•1HCl•0.3H_2O: C, 42.14; H, 7.43; N, 7.94. Found: C, 41.95; H, 7.04; N, 7.94.

Compound 4b. Compound **4b** (4.2 mg, 23 μmol, white solid, 94%) was obtained from **9b** (8.3 mg, 25 μmol) as described for the preparation of **4a** (Figure 4). $[\alpha]_D^{16}$ = +6.76° (c 0.28, CH_3OH); ^{1}H NMR (500 MHz, CD_3OD) δ 2.64 (1 H, dd, J = 5.5, 8.5 Hz), 2.57 (1 H, d, J = 20.5 Hz), 2.10 (1 H, d, J = 20.5 Hz), 1.74 (1 H, m), 1.38 (1 H, m), 1.08 (3 H, t, J = 9.5 Hz), 0.99 (1 H, t, J = 8.5 Hz), 0.71 (1 H, dd, J = 5.5, 8.5 Hz); ^{13}C NMR (125 MHz, CD_3OD) δ 174.9, 39.5, 34.5, 23.7, 23.5, 16.8, 10.9; LRMS (ESI) m/z 144 [(M+H)$^+$]; Anal. calcd for $C_7H_{13}NO_2$•1HCl•0.1H_2O: C, 46.34; H, 7.89; N, 7.72. Found: C, 46.11; H, 7.74; N, 7.59.

Compound 4c. Compound **4c** (11 mg, 48 μmol, white solid, 97%) was obtained from **9c** (26 mg, 96 μmol) as described for the preparation **4a** (Figure 4). $[\alpha]_D^{16}$ = +2.37° (c 0.38, CH_3OH); ^{1}H NMR (500 MHz, CD_3OD) δ 7.50–7.33 (5 H, m), 2.97 (1 H, m), 2.79 (1 H, d, J = 16.0 Hz), 2.49 (1 H, d, J = 16.0 Hz), 1.49 (1 H, m), 1.41 (1 H, m); ^{13}C NMR (125 MHz, CD_3OD) δ 174.1, 137.6, 131.7, 130.2, 129.4, 44.9, 33.2, 28.6, 16.8; LRMS (ESI) m/z 192 [(M+H)$^+$]; Anal. calcd for $C_7H_{13}NO_2$•1HCl•0.4H_2O: C, 56.25; H, 6.35; N, 5.96. Found: C, 56.22; H, 6.03; N, 5.93.

References

Hoshiya, N., Kobayashi, T., Arisawa, M., and Shuto, S. (2013). Palladium-Catalyzed Arylation of Cyclopropanes via Directing Group-Mediated C(sp3)–H Bond Activation To Construct Quaternary Carbon Centers: Synthesis of cis- and trans-1,1,2-Trisubstituted Chiral Cyclopropanes. *Org. Lett.* 15, 6202–5. doi:10.1021/ol4030452.

Hoshiya, N., Takenaka, K., Shuto, S., and Uenishi, J. (2016). Pd(II)-Catalyzed Alkylation of Tertiary Carbon via Directing-Group-Mediated C(sp3)–H Activation: Synthesis of Chiral 1,1,2-Trialkyl Substituted Cyclopropanes. *Org. Lett.* 18, 48–51. doi:10.1021/acs.orglett.5b03229.

Kickinger, S., Hellsberg, E., Frølund, B., Schousboe, A., Ecker, G. F., and Wellendorph, P. (2019). Structural and molecular aspects of betaine-GABA transporter 1 (BGT1) and its relation to brain function. *Neuropharmacology* 161, 107644. doi:10.1016/j.neuropharm.2019.05.021.

Suemasa, A., Watanabe, M., Kobayashi, T., Suzuki, H., Fukuda, H., Minami, M., et al. (2018). Design and synthesis of cyclopropane-based conformationally restricted GABA analogues as selective inhibitors for betaine/GABA transporter 1. *Bioorg. Med. Chem. Lett.* 28, 3395–3399. doi:10.1016/j.bmcl.2018.08.031.

Yamashita, A., Singh, S. K., Kawate, T., Jin, Y., and Gouaux, E. (2005). Crystal structure of a bacterial homologue of Na^+/Cl^--dependent neurotransmitter transporters. *Nature* 437, 215–23. doi:10.1038/nature03978.
